# ONECUT transcription factors induce neuronal characteristics and remodel chromatin accessibility

**DOI:** 10.1093/nar/gkz273

**Published:** 2019-05-03

**Authors:** Jori van der Raadt, Sebastianus H C van Gestel, Nael Nadif Kasri, Cornelis A Albers

**Affiliations:** 1 Department of Human Genetics, Donders Institute for Brain, Cognition and Behavior, Radboud University Medical Center, Nijmegen, The Netherlands; 2 Department of Molecular Developmental Biology, Radboud Institute for Molecular Life Sciences, Radboud University, Nijmegen, The Netherlands; 3 Department of Cognitive Neuroscience, Donders Institute for Brain, Cognition and Behavior, Radboud University Medical Center, Nijmegen, The Netherlands

## Abstract

Remodeling of chromatin accessibility is necessary for successful reprogramming of fibroblasts to neurons. However, it is still not fully known which transcription factors can induce a neuronal chromatin accessibility profile when overexpressed in fibroblasts. To identify such transcription factors, we used ATAC-sequencing to generate differential chromatin accessibility profiles between human fibroblasts and iNeurons, an *in vitro* neuronal model system obtained by overexpression of Neurog2 in induced pluripotent stem cells (iPSCs). We found that the ONECUT transcription factor sequence motif was strongly associated with differential chromatin accessibility between iNeurons and fibroblasts. All three ONECUT transcription factors associated with this motif (ONECUT1, ONECUT2 and ONECUT3) induced a neuron-like morphology and expression of neuronal genes within two days of overexpression in fibroblasts. We observed widespread remodeling of chromatin accessibility; in particular, we found that chromatin regions that contain the ONECUT motif were in- or lowly accessible in fibroblasts and became accessible after the overexpression of ONECUT1, ONECUT2 or ONECUT3. There was substantial overlap with iNeurons, still, many regions that gained accessibility following ONECUT overexpression were not accessible in iNeurons. Our study highlights both the potential and challenges of ONECUT-based direct neuronal reprogramming.

## INTRODUCTION

Reprogramming of somatic cells directly into neurons has previously been achieved by overexpression of transcription factors (TFs) (1–3) and by TFs in combination with microRNA’s (4,5). Small molecules can induce neuronal reprogramming on their own (6,7) or can significantly enhance reprogramming efficiency when combined with TFs or microRNAs (8,9). Direct neuronal reprogramming has important potential applications in personalized medicine and cell replacement therapy (10,11).

Chromatin accessibility is a key feature of cell type identity. Accessible chromatin, or open chromatin regions (OCRs), are highly cell type-specific and are strongly correlated with where TFs bind to the DNA (12). TF DNA binding motifs associated with differentially accessible chromatin are predictive of cell-type specific gene expression (13). Multiple studies have shown that chromatin accessibility is remodeled during direct neuronal reprogramming (14–16). One of the most potent neuronal reprogramming factors, Ascl1, acts as a pioneer factor by binding to its target sequence in closed chromatin and induces widespread chromatin changes within twelve hours after induction (14,17). Moreover, the combination of mir-9/9* and mir-124 remodels the chromatin accessibility towards a neuronal state by changing the BAF complex (an ATP-dependent chromatin remodeling complex (18)) into a neuron-specific composition (15). Small molecules that enhance chromatin accessibility have been shown to enhance Neurog2-based neuronal conversion of fibroblasts to motor neurons (16).

In general, however, the TFs that can induce chromatin accessibility associated with neurons are not fully known. Here, our aim was to identify additional TFs that can induce chromatin accessibility associated with neurons when overexpressed in fibroblasts. It has previously been shown that overexpression of Neurog2 differentiates human induced pluripotent stem cells (hiPSCs) into functional neurons (iNeurons) (19). Here, we used iNeurons as an *in vitro* neuronal model system. We generated ATAC-seq profiles for iNeurons and human fibroblasts and used ATAC-seq fragment count as a proxy for chromatin accessibility. We found that ONECUT1, ONECUT2 and ONECUT3 were the TFs most strongly associated with differential chromatin accessibility, and that individual overexpression of these TFs in fibroblasts resulted in induction of neuronal characteristics and rapid remodeling of chromatin accessibility within 2 days.

## MATERIALS AND METHODS

### Cell culture

The fibroblasts lines (Supplementary Table S1) were cultured in tissue culture flasks (Corning) in Dulbecco's modified Eagle's medium containing 20% (vol/vol) fetal bovine serum, 1% (vol/vol) penicillin/streptomycin and 1% (vol/vol) sodium pyruvate (all from Sigma-Aldrich), from here on referred to as fibroblast medium. iPSC lines were obtained by lentiviral transduction of two of the fibroblast lines with the mouse OSKM (Oct4, Sox2, Klf4, Myc) cocktail. iPSC lines were cultured in 6 well plates coated with vitronectin (Gibco) in E8 medium (Gibco) containing 50 μg/ml G418 (Sigma-Aldrich) and 0.5 μg/ml puromycin (Sigma-Aldrich).

### iNeuron differentiation

iNeuron differentiation was performed as described previously (20). Briefly, rtTA/Neurog2-positive iPSC lines were differentiated to iNeurons via doxycyclin-dependent Neurog2 overexpression over a period of three weeks (19). On day 21 after induction, cells were isolated for ATAC-seq and RNA-seq. Supplementary Table S2 details on the rtTA and Neurog2 transfer vectors.

### Validation experiments

The validation experiments consisted of overexpressing OC1/2/3 in human adult skin fibroblasts and were performed as follows. On day –2, 20 000 fibroblasts were plated in 1 ml fibroblast medium in each well of a twelve wells plate (Corning). On day –1, cells were transduced with either only the Bclxl, OC1, OC2 or OC3 vector or the Bclxl vector in combination with the OC1, OC2 or OC3 vector (Supplementary Table S2). Transduction was performed in fresh fibroblast medium in the presence of 8ug/ml polybrene (Sigma-Aldrich). On day 0, 2 and 4, the medium was refreshed for medium containing 2 ug/ml doxycycline (Sigma-Aldrich) to induce expression of the OC1/2/3 transgene. For ATAC-seq and RNA-seq, cells were isolated on day 2.

### Immunohistochemistry

Cells were fixed in ice-cold 4% (mass/vol) paraformaldehyde in PBS, permeabilized with 0.2% Triton X-100 in PBS, incubated with blocking buffer consisting of 10% (vol/vol) goat serum in PBS, incubated with the primary antibodies rabbit anti-ONECUT3 (Biorbyt, orb312423, 1:100) and mouse anti-TUBB3 (Covance, MMS-435P, 1:1000) in 5% (vol/vol) goat serum in PBS at 4°C overnight, incubated with the conjugated secondary antibodies (Invitrogen) in 5% (vol/vol) goat serum in PBS at RT for 1 h, incubated with 0.01% (vol/vol) Hoechst (Invitrogen) in PBS at RT for 10 min, and finally mounted onto slides with fluorescence mounting medium (Dako). Imaging was performed using an Axio Imager Z1 (Zeiss). CellProfiler (21) was used to quantify staining intensity for individual cells at the nucleus.

### ATAC-sequencing

In short, nuclei were isolated by adding 500 μl lysis buffer (10 mM Tris/HCl [pH 7.5], 10 mM NaCl, 3 mM MgCl_2_ and 0.2% [vol/vol] IGEPAL, in Milli-Q water/PBS [1:1]) to the cell pellet (iPSCs and fibroblasts) or to the cells on the plate (iNeurons), followed by mechanically dissociating the cells using a pipette. Nuclei were pelleted, washed, and incubated with the Tn5 transposase in a shaking heat block at 37°C/650 rpm for 1 h. The reaction was stopped by adding 5 μl clean-up buffer (0.9 M NaCl and 0.3 M EDTA [pH 8] in nuclease-free water), 2 μl proteinase K (10 mg/ml) and 2 μl 5% SDS. DNA was purified using normal-phase 2× AMPure bead purification (Beckman Coulter), PCR amplified for 8 PCR cycles, size-selected using reverse-phase 0.55× AMPure bead purification, purified using a Qiaquick column (Qiagen), PCR amplified for another eight PCR cycles, and again purified on column. Sequencing was performed on an Illumina NextSeq 500 using HighOutput kit v2 for 75 cycles (paired-end 2 × 43 bp). The number of mapped ATAC-seq reads can be found in Supplementary Table S3.

### RNA-sequencing

RNA was isolated using the RNeasy Mini kit (Qiagen) and RNA-seq library preparation was performed using the SMARTer Stranded Total RNA Sample Prep Kit (low input mammalian) (Clontech). Sequencing was performed on an Illumina NextSeq 500 using HighOutput kit v2 for 75 cycles (paired-end 2 × 43 bp). The number of mapped RNA-seq reads can be found in Supplementary Table S3.

### Union CR sets

We used three different union CR sets:

1. iNeuron-Fibroblast union: A reduced union CR set made by combining the iNeuron and fibroblast MACS2 peaksets and merging overlapping peaks to one peak.
2. ATAC union: A reduced union CR set made by combining the Bclxl, OC1+Bclxl, OC2+Bclxl, OC3+Bclxl, fibroblast, iPSC and iNeuron MACS2 peaksets and merging overlapping peaks to one peak.
3. DNase union: A union set of CRs with DNase I accessibility in at least one of 127 ENCODE/ROADMAP cell types.

### Differential chromatin accessibility and gene expression

Both differential chromatin accessibility and differential gene expression were determined using DESeq2 (22). As input we used count values from the ATAC-seq/RNA-seq for CRs/genes. In all differential analyses with DESeq2, we used the design: *design ∼ cell line + cell type*, to take into account both cell line and cell type. We considered CRs/genes differentially upregulated if the Benjamini-Hochberg-adjusted *P*-value < 0.01 and the log_2_(fold change) > 1. Similarly, we considered CRs/genes differentially downregulated if the Benjamini-Hochberg-adjusted *P*-value < 0.01 and the log_2_(fold change) < –1.

Using these criteria, we defined the following sets of differential accessible CRs: more accessible in OC1+Bclxl, OC2+Bclxl or OC3+Bclxl compared to Bclxl as respectively *OC1-up, OC2-up* and *OC3-up*; less accessible in OC1+Bclxl, OC2+Bclxl or OC3+Bclxl compared to Bclxl as respectively *OC1-down, OC2-down* and *OC3-down*; more accessible in iNeurons than in fibroblast as iN-Fib up; less accessible in iNeurons than in fibroblasts as iN-Fib down. We defined the differentially expressed gene sets in the same way.

Detailed Materials and Methods can be found in the supplementary materials.

## RESULTS

### The CUX and ONECUT transcription factor motifs are strongly associated with differential chromatin accessibility

**Figure 1.**
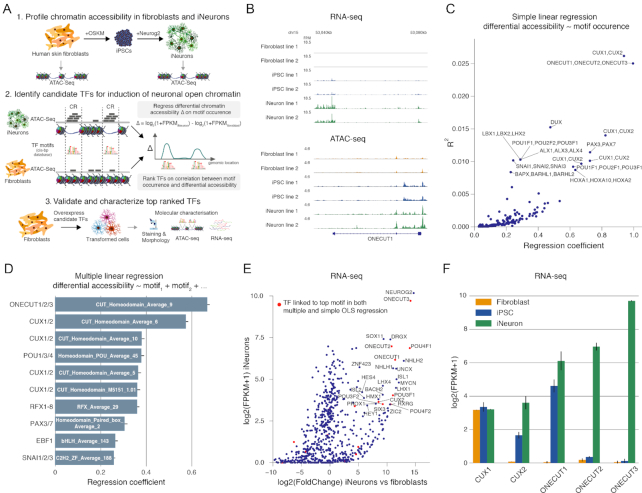
Identification of transcription factors associated with differential chromatin accessibility between human iNeurons and fibroblasts. (**A**) Overview of approach. (i) Chromatin accessibility in iPSC-derived iNeurons and fibroblasts was experimentally measured with ATAC-Seq. (ii) Differential chromatin accessibility (based on normalized ATAC-seq fragment count, see Methods) between iNeurons and fibroblasts was correlated with predicted transcription factor (TF) motif occurrence (based on cis-bp database (26)) to identify candidate TFs that could induce of iNeuronal OCRs when overexpressed in fibroblasts. (iii) The candidate TFs were validated and characterized. (**B**) Representative example of RNA-seq and ATAC-seq data used for identifying candidate TFs. Only one of two replicates per cell line is shown. FPM, fragments per million. (**C**) Simple linear regression of differential chromatin accessibility between iNeurons and fibroblasts on the TF motif occurrence for 580 motifs. The figure shows variance explained (*R*^2^) versus regression coefficient. Each point represents a separate regression. (**D**) Multiple linear regression of differential chromatin accessibility between iNeurons and fibroblasts on all 580 motifs simultaneously. The multiple regression identifies the effect of a TF motif on differential accessibility that cannot be explained by the other motifs. The figure shows the regression coefficient and its standard error. (**E**) Gene expression (RNA-seq) for the TFs that are linked to the motifs used for the ATAC-seq regression analysis. (**F**) Gene expression in fibroblasts, iPSCs and iNeurons for the top candidates from the ATAC-seq regression analysis. Plotted is the log_2_(1+FPKM) quantile normalized gene expression for CUX1, CUX2, ONECUT1, ONECUT2 and ONECUT3.

Our strategy to identify candidate chromatin remodeling TFs is summarized in Figure 1A. Briefly, we profiled chromatin accessibility using Assay for Transposase-Accessible Chromatin using sequencing (ATAC-seq) (23) for two human fibroblast lines from different individuals, iPSCs derived from these fibroblasts, and iNeurons derived from these iPSCs by overexpression of Neurog2 for 21 days (Figure 1A, Supplementary Figure S1), as we described previously (20). We used the MACS2 peak caller (24) to identify open chromatin regions (OCRs) using the ATAC-seq data of two replicates for each cell line. A union chromatin region (CR) set was constructed from the fibroblast and iNeuron OCRs (‘iNeuron-Fibroblast union’), merging overlapping OCRs. Thus, a CR from the union set may be accessible in one cell type but not in another. To identify TFs associated with differential chromatin accessibility, we regressed differential chromatin accessibility between iNeurons and fibroblasts at iNeuron-Fibroblast union CRs, defined as Δ = log_2_(1+FPKM_iNeuron_) – log_2_(1+FPKM_fibroblast_), on the presence of a TF motif M using a simple linear regression model: $\Delta \ = \ \mu + \beta M + \epsilon$ (see Materials and Methods). We used GimmeMotifs (25) to generate for 580 clustered TF motifs, based on the *cis*-bp database (26), binary motif calls with *M* = 1 to encode the presence of a motif in a particular CR and *M* = 0 to encode the predicted absence of a motif in a CR.

The simple linear regression identified the ONECUT TF family associated motif CUT_Homeodomain_Average_9, and the CUX TF family associated motif CUT_Homeodomain_Average_6, as the motifs most predictive of chromatin that is more open in iNeurons than in fibroblasts (regression coefficients β of resp. 0.99, 0.94; variance explained *r*^2^ of resp. 0.025, 0.026, *P*-value < 10^−16^; Figure 1C). Even though similar motifs were clustered prior to the regression, residual similarity between motifs complicate interpretation of the simple linear regression (Supplementary Figure S2). Therefore, in order to evaluate the contribution of a motif that cannot be explained by similarity with other motifs, we also performed a multiple linear regression that predicted differential chromatin accessibility using all 580 motifs simultaneously: $\Delta \ = \ \mu + {\beta _1}{M_1} + {\beta _2}{M_2} + \ldots + {\beta _{580}}{M_{580}} + \epsilon .$ We found that the CUT_Homeodomain_Average_9 and CUT_Homeodomain_Average_6 motifs were also most predictive when controlling for similarity with other motifs (Figure 1D).

Three distinct TFs, ONECUT1 (OC1), ONECUT2 (OC2) and ONECUT3 (OC3), are associated with the CUT_Homeodomain_Average_9 motif and two TFs, CUX1 and CUX2, are associated with the CUT_Homeodomain_Average_6 motif. We generated RNA-sequencing (RNA-seq) data for the same biological samples to determine whether a complete lack of gene expression may be used to rule out one of these TFs as binding to the motif in iNeurons. This was not the case: all three ONECUT TFs were among the highest expressed TFs in iNeurons and were not expressed in fibroblasts (Figure 1E and F). CUX1 was expressed at similar levels in fibroblasts, iPSCs and iNeurons, while CUX2 was only expressed in iPSCs and iNeurons. Thus, in iNeurons, all five TFs potentially bind to their associated DNA sequence motif.

We selected OC1, OC2 and OC3 (OC1/2/3) as candidate chromatin remodeling factors for further experimental validation and characterization.

### OC1, OC2 and OC3 induce neuron-like morphology, TUBB3 expression and cell death when overexpressed in fibroblasts

**Figure 2.**
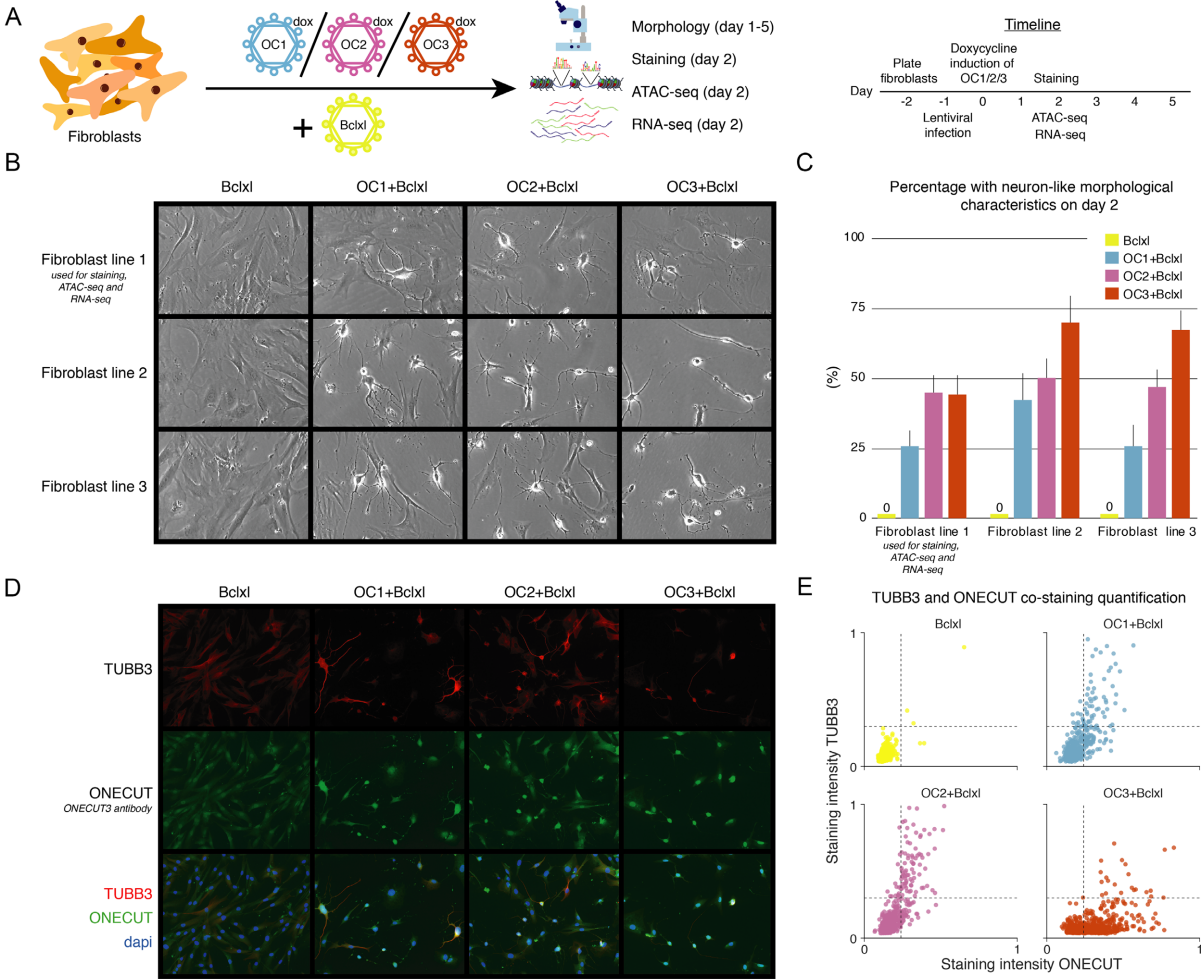
Phenotypic effects of ONECUT overexpression in fibroblasts. (**A**) Outline validation experiments. Human fibroblasts were lentivirally transduced with the ONECUT1 (OC1), ONECUT2 (OC2) or ONECUT3 (OC3) transgene together with the anti-apoptotic transgene Bclxl. We assessed morphology, neuronal marker expression, ATAC-seq and RNA-seq. (**B**) Cell morphology on day 2 after induction of OC1/2/3 overexpression in three different adult human fibroblast cell lines overexpressing Bclxl. Fibroblast line 1 was used for stainings, ATAC-seq and RNA-seq. (**C**) Percentage of cells with neuron-like morphological characteristics two days after OC1/2/3 induction for all three fibroblast lines. (**D**) Co-staining for TUBB3 and ONECUT (anti-ONECUT3 antibody, see methods) 2 days after OC1/2/3 induction in fibroblast line 1. (**E**) Quantification of the mean staining intensity at individual nuclei for TUBB3 and ONECUT. Shown are 668 nuclei per condition. The horizontal and vertical dotted lines are there to help compare OC1+Bclxl, OC2+Bclxl and OC3+Bclxl to the Bclxl condition.

We used a doxycycline-inducible lentiviral construct to overexpress the ONECUT factors individually in three human fibroblast lines (Figure 2A). Previous studies have shown that overexpression of the anti-apoptotic gene Bclxl reduces cell death in the context of direct neuronal reprogramming (16,27). To reduce the potential toxic effect of OC1/2/3 overexpression, we therefore co-overexpressed each ONECUT factor with Bclxl (see Methods). The titers used for all experiments are shown in Supplementary Figure S3a. With these titers, OC1/2/3 were overexpressed at a higher level than their endogenous expression in iNeurons (Supplementary Figure S3b).

We found that OC1/2/3 overexpression induced a neuron-like morphology as early as two days after induction in fibroblast medium (Figure 2B), both with and without co-expression of Bclxl (Supplementary Figure S4A–C). In combination with Bclxl, neuron-like morphological characteristics were induced in 25%-69% of the fibroblasts (Figure 2C, Supplementary Figure S5A and B). OC1/2/3 overexpression also enhanced the expression of the neuronal marker TUBB3 in a subset of cells (Figure 2C, D, Supplementary Figures S6, S7A–C). However, OC1/2/3 overexpression also resulted in extensive cell death and few cells with a neuron-like morphology survived for more than five days after induction (Supplementary Figure S4A–C). Overexpression of Bclxl alone did not change the fibroblast morphology or upregulate TUBB3 expression (Supplementary Figure S7C and D).

### Chromatin accessibility is extensively remodeled after OC1, OC2 or OC3 overexpression, and is increased at CRs with low accessibility in fibroblasts

**Figure 3.**
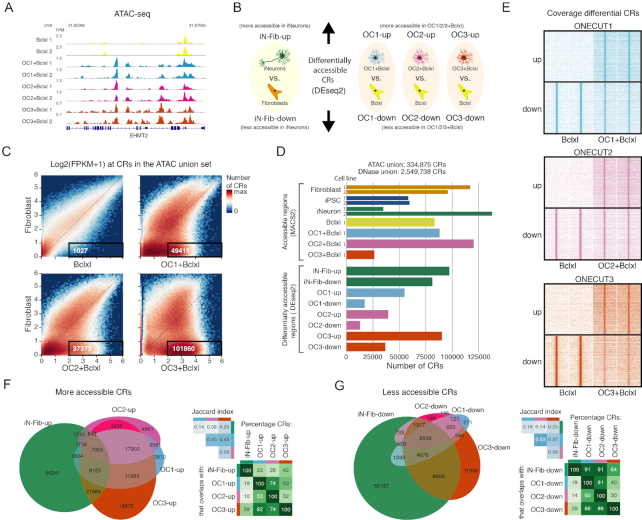
Chromatin accessibility changes induced by overexpression of ONECUT factors in fibroblasts. (**A**) Representative example of the ATAC-seq data on day 2 after induction of ONECUT transgene expression. FPM, fragments per million. (**B**) Definition of sets of differentially accessible chromatin regions (CRs), as determined by DESeq2 using ATAC-seq fragment counts at ATAC union CRs. (**C**) 2D histogram of log2-transformed ATAC-seq fragment counts (without quantile normalization) at the ATAC union CRs, comparing fibroblasts with respectively, the Bclxl, OC1+Bclxl, OC2+Bclxl and OC3+Bclxl conditions. Boxes indicate CRs with log2(1+FPKM) < 1 in fibroblasts and log2(1+FPKM) > 2 in respectively the Bclxl, OC1+Bclxl, OC2+Bclxl or OC3+Bclxl condition. (**D**) Number of CRs in the union CR sets, MACS2 peak sets and differentially accessible CR sets. Where applicable, biological replicates (cell lines from different individuals) are plotted separately. Technical replicates were jointly analyzed by MACS2 to produce a single ATAC-seq CR et for each cell line. (**E**) RPKM normalized ATAC-seq coverage at differential CRs. Heatmaps of ATAC-seq coverage showing 10 kb windows centered on CRs differentially accessible following ONECUT1, ONECUT2 or ONECUT3 overexpression. For each CR, two technical replicates for both the Bclxl and the OC1/2/3+Bclxl condition are shown. (**F**) Venn diagram, Jaccard indices and overlap percentages for the overlap between differentially more accessible CR sets (top row in panel B). Venn diagram areas are approximately proportional to the number of CRs. (**G**) Venn diagram, Jaccard indices and overlap percentages, for the overlap between differentially less accessible CR sets (bottom row in panel B). Venn diagram areas are approximately proportional to the number of CRs.

We next investigated whether the morphological changes were accompanied by changes in chromatin accessibility. We characterized chromatin accessibility with ATAC-seq two days after induction of OC1/2/3. As a control, we performed ATAC-seq of fibroblasts only overexpressing Bclxl (Figure 3A). We used the MACS2 peak caller (24) to identify OCRs, using the ATAC-seq data of two replicates for each condition (see Methods). We constructed a union CR set (‘ATAC union’) from the OC1+Bclxl, OC2+Bclxl, OC3+Bclxl, Bclxl, fibroblast, iPSC and iNeuron OCRs, merging overlapping OCRs. ATAC-seq FPKM values were quantified on this ATAC union CR set. With ATAC-seq fragment count as a proxy for chromatin accessibility, we used DESeq2 (22) to identify CRs that became significantly more accessible (upregulated) or less accessible (downregulated) after overexpression of a ONECUT factor. We defined four categories of differentially accessible CRs (Figure 3B): (i) CRs that were more accessible following OC1, OC2 or OC3 overexpression in fibroblast as respectively *OC1-up, OC2-up* and *OC3-up*; (ii) CRs that were less accessible after OC1, OC2 or OC3 overexpression in fibroblasts as respectively *OC1-down, OC2-down* and *OC3-down*; (iii) CRs that were more accessible in iNeurons than in fibroblast as *iN-Fib-up*; (iv) and CRs less accessible in iNeurons than in fibroblasts as *iN-Fib-down*.

We found that chromatin accessibility was extensively remodeled following OC1/2/3 overexpression (Figure 3C). Respectively 49 415, 37 373 and 101 860 CRs with low accessibility (log2(1+FPKM)<1) in fibroblasts had higher accessibility (log2(1+FPKM)>2) following overexpression of OC1, OC2 and OC3 (Figure 3C, Supplementary Figure S8). The corresponding number of CRs was 1027 for the overexpression of Bclxl alone (Figure 3C, Supplementary Figure S8). When quantified with DESeq2, thousands of CRs had significantly altered chromatin accessibility after OC1/2/3 overexpression (Figure 3D); upregulated CRs identified by DESeq2 indeed had low accessibility in fibroblasts overexpressing Bclxl (Figure 3E).

To investigate to what extent the changes in chromatin accessibility are shared between OC1, OC2 and OC3 overexpression, we intersected the differentially accessible CR sets. Many of the differentially accessible CRs were shared between the three ONECUT factors: for instance, 82% of regions upregulated by OC1 (*OC1-up*) were also upregulated by OC3; vice versa, 50% of regions (*OC3-up*) upregulated by OC3 were also upregulated by OC1 (Figure 3F). Similarly, many downregulated regions (*OC1-down, OC2-down, OC3-down*) were also shared between the ONECUT factors (Figure 3G).

### The ONECUT motif is strongly associated with CRs that gain accessibility following OC1/2/3 overexpression in fibroblasts

To gain more insight into the mechanism by which OC1/2/3 overexpression remodels chromatin accessibility, we investigated the sequence specificity of changes in accessibility. More specifically, we asked to what extent the ONECUT motif (Supplementary Figure S2) is associated with the observed differential accessibility.

**Figure 4.**
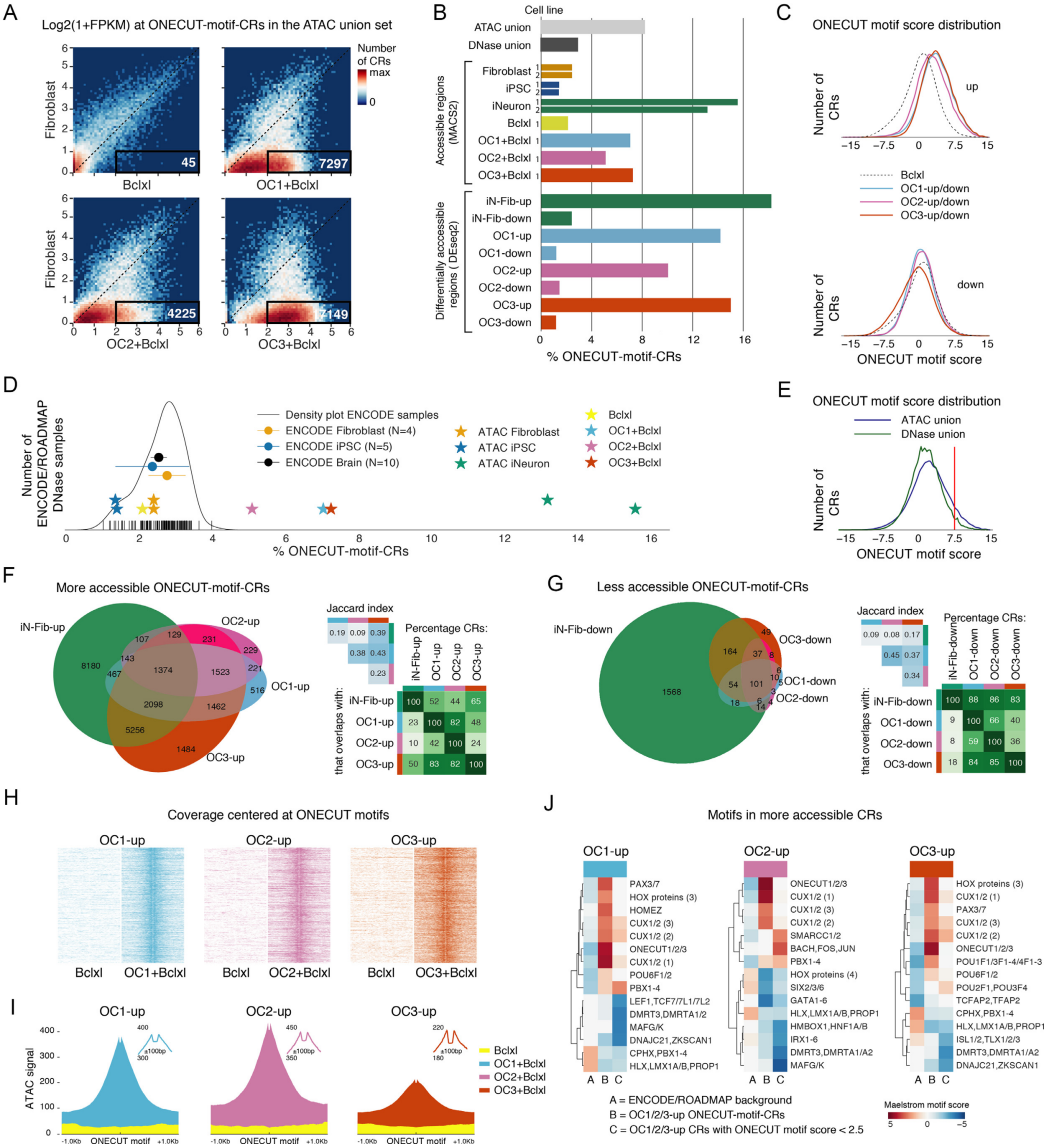
Chromatin accessibility changes induced by overexpression of ONECUT factors in fibroblasts at chromatin regions containing the ONECUT sequence motif. (**A**) 2D histogram of log2-transformed ATAC-seq fragment counts (without quantile normalization) at the ATAC union CRs containing the ONECUT sequence motif. This panel is the equivalent of Figure 3C, restricted to the subset ONECUT-motif-CRs. Boxes indicate CRs with log2(1+FPKM) < 1 in fibroblasts and log2(1+FPKM) > 2 in respectively the Bclxl, OC1+Bclxl, OC2+Bclxl or OC3+Bclxl condition. (**B**) Percentage of CRs containing the ONECUT sequence motif in union CR sets, peak sets (MACS2) and differentially accessible CR sets (DESeq2). (**C**) ONECUT motif score distribution across CRs called by MACS2 for the Bclxl condition, and in the subsets of respectively more accessible CRs (*OC1/2/3-up*) and less accessible CRs (*OC1/2/3-down*) following OC1/2/3 overexpression. (**D**) Comparison of the percentage of ONECUT-motif-CRs for the ATAC-seq MACS2 CR sets and 53 DNase I sequencing samples from the ENCODE/ROADMAP projects. We restricted ATAC-seq CRs to those that overlap with an ENCODE/ROADMAP DNase Hypersensitivity Site (DHS) in at least one DNase sample, and resized all CRs to a width of 200 bp. The density plot is across all DNase samples. Circles indicate means and standard deviations for the percentage of DHSs that contain the ONECUT motif for selected groups of DNase samples; stars indicate the percentage of CRs containing the ONECUT motif for individual biological replicates from this study. (**E**) ONECUT motif score distribution across all ATAC-seq CRs (ATAC union) and all ENCODE/ROADMAP DHS (DNase union). The dotted red line indicates the threshold that was used to determine ONECUT-motif-CRs (CRs with a ONECUT motif score > 7.5). (**F**) Venn diagram, Jaccard indices and overlap percentages for the overlap between differentially more accessible CR sets (top row Figure 3b), restricted to ONECUT-motif-CRs. Venn diagram areas are approximately proportional to the number of CRs. (**G**) Venn diagram, Jaccard indices and overlap percentages for the overlap between differentially less accessible CR sets (bottom row Figure 3B), restricted to ONECUT-motif-CRs. Venn diagram areas are approximately proportional to the number of CRs. (**H**) RPKM normalized ATAC-seq heatmaps of fragment count per base pair, showing 2 kb windows centered on the ONECUT motifs in the OC1-up, OC2-up or OC3-up CR set. For each condition plotted, the reads of two technical replicates were combined. (**I**) Average RPKM normalized ATAC-seq fragment count per base pair centered at ONECUT motif in in the OC1-up, OC2-up or OC3-up CR set. A 2 kb window, averaged over the individual ONECUT motifs. For each condition plotted, the reads of two technical replicates were combined. (**J**) Enriched motifs in (ONECUT-motif-)CRs more accessible following OC/1/2/3 overexpresssion. Motifs determined using the GimmeMotifs function maelstrom, comparing an ENCODE/ROADMAP background CR set (A) to more accessible CRs with a ONECUT motif (B) and more accessible CRs without a ONECUT motif (C). Motif logos in Supplementary Figure S9.

CRs that contain a ONECUT motif (ONECUT-motif-CRs) gained accessibility following OC1/2/3 overexpression (Figure 4A). 21 599 (78%) of the 27 794 ONECUT-motif-CRs in the ATAC union set had low accessibility (defined as log2(FPKM+1) < 1) in fibroblasts (Figure 4A). Respectively 7297, 4225 and 17 149 ONECUT-motif-CRs with low accessibility in fibroblasts had increased accessibility (defined as log_2_(FPKM+1) > 2) following overexpression of respectively OC1, OC2, OC3 (Figure 4A). In contrast, there were only 45 such ONECUT-motif-CR for overexpression of Bclxl alone (Figure 4A). Thus, overexpression of each of the ONECUT factors resulted in increased chromatin accessibility at previously in- or lowly accessible ONECUT-motif-CRs.

To further quantify the association between CRs that gained accessibility after OC1/2/3 overexpression and the ONECUT motif, we calculated the percentage of CRs that contained a ONECUT motif in the various CR sets (Figure 3D). iNeuron biological replicates had a high percentage (15.6% and 13.2%) of ONECUT-motif-CRs compared to fibroblasts (2.4% and 2.5%) and iPSC (1.4% and 1.4%) (Figure 4B). CRs that were significantly more accessible after OC1/2/3 overexpression were enriched for the ONECUT motif, with 14.2%, 10.0% and 15.0% of respectively *OC1-up, OC2-up* and *OC3-up* CRs containing a ONECUT motif (Figure 4B). Consistent with these observations, the ONECUT-motif-score distribution for CRs that gained accessibility was shifted towards higher scores relative to fibroblasts overexpressing Bclxl (Figure 4C). The fraction of ONECUT-motif-CRs in iNeurons and OC1/2/3 conditions were high compared to the cell types and tissues present in 53 ENCODE/ROADMAP DNase I hypersensitivity sequencing (DNase-seq) samples (Figure 4D and E) (28,29).

To determine, specifically at ONECUT-motif-CRs, to what extent the changes in chromatin accessibility are shared between OC1, OC2 and OC3 overexpression, we overlapped the differentially accessible ONECUT-motif-CR sets. Also when restricted to the subset of ONECUT-motif-CRs, there was substantial overlap between CRs that were differentially accessible following OC1/2/3 overexpression (Figure 4F and G).

Footprints in ATAC-seq data are signatures of TF binding (23). To investigate the mechanism by which OC1/2/3 overexpression enhances chromatin accessibility, we centered the ATAC-seq coverage at ONECUT motifs in *OC1/2/3-up* CRs. At these CRs, a valley is visible in the average ATAC-seq signal at ONECUT motifs (Figure 4H and I). This suggests that a protein is binding to the ONECUT DNA motif, which prevents the Tn5 transposase from accessing the DNA.

To identify other TFs that might be involved in enhancing chromatin accessibility following OC1/2/3 overexpression, we searched for enriched motifs in the *OC1-up, OC2-up* and *OC3-up* CRs. We distinguished ONECUT-motif-CRs and CRs with a ONECUT motif score <2.5, and compared these a background CR set. We found that *OC1/2/3-up* ONECUT-motif-CRs were enriched for the ONECUT motif and very similar AT-rich motifs (Figure 4J, Supplementary Figures S9 and S10A). *OC1-up* and *OC2-up* CRs with a low ONECUT motif score were enriched for a motif associated with PBX1, PBX2, PBX3 and PBX4 (Figure 4J, Supplementary Figures S9 and S10a). PBX1–4 were all expressed following OC1/2/3 overexpression and PBX4 was upregulated (Supplementary Figure S10B).

### Comparison of chromatin accessibility in iNeurons and ONECUT overexpression conditions

Given that the ONECUT motif was strongly associated with differential accessibility between iNeurons and fibroblasts, a key question is if overexpression of OC1/2/3 in fibroblasts makes chromatin accessibility more similar to that of iNeurons.

**Figure 5.**
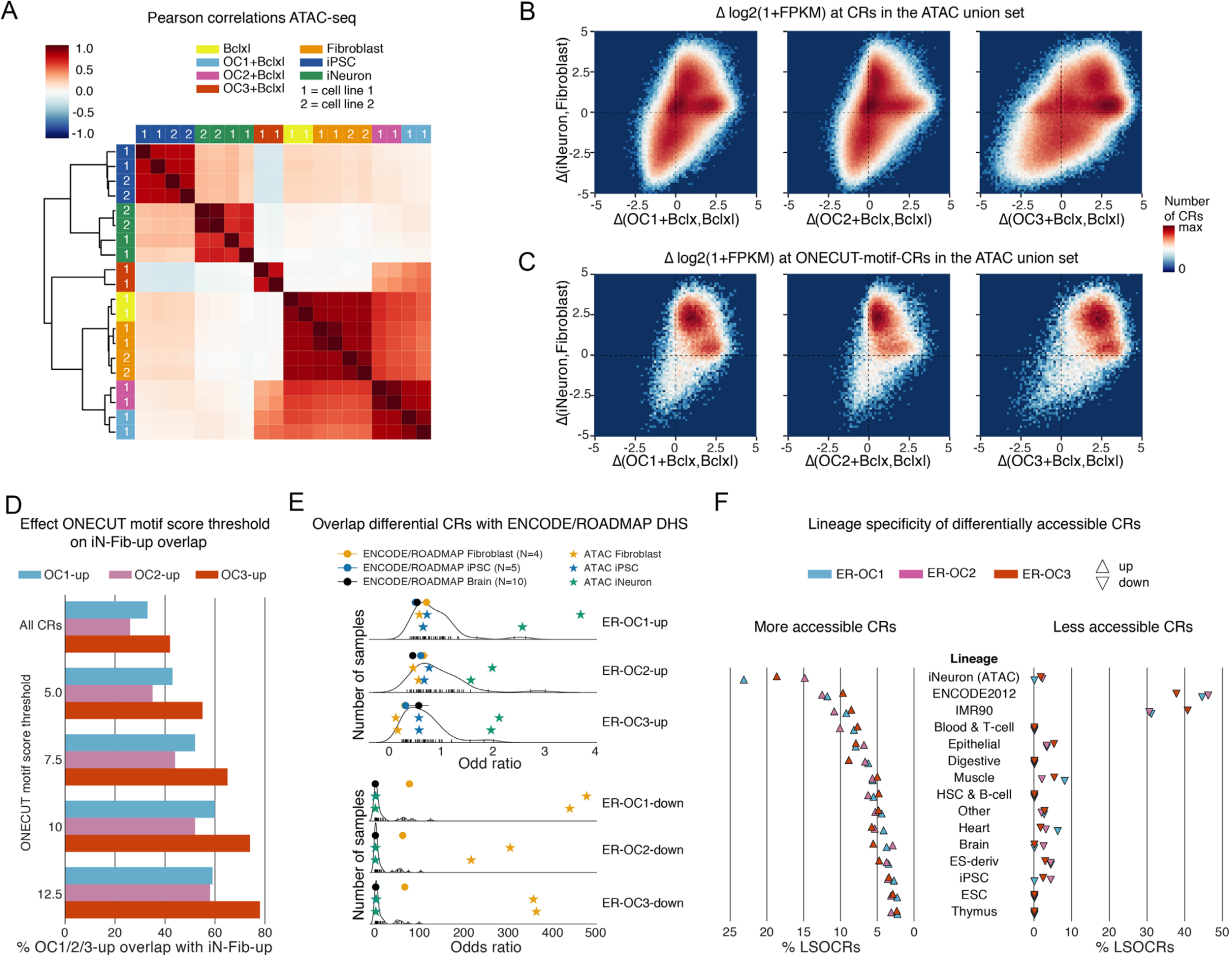
Comparison of ONECUT factor induced differential chromatin accessibility with differential chromatin accessibility between iNeurons and fibroblasts. (**A**) Hierarchically clustered heatmap of Pearson correlation coefficients of the quantile normalized log_2_-transformed ATAC-seq fragment counts to visualize overall similarity between conditions. Row and column colors indicate cell type, numbers inside the row and column colors indicate cell line. (**B**) 2D histogram of differences in chromatin accessibility for cell line 1 at ATAC union CRs. Differences between iNeurons and fibroblasts (ΔiNeuron,Fibroblast) on the y-axis. Differences between OC1+Bclxl (ΔOC1+Bclxl,Bclxl), OC2+Bclxl (ΔOC2+Bclxl,Bclxl) or OC3+Bclxl (ΔOC1+Bclxl,Bclxl) and Bclxl on the x-axis. Δ is the difference in log2-transformed ATAC-seq fragment count, averaged across technical replicates. (**C**) Identical to panel B but restricted to the ONECUT-motif-CRs. (**D**) Overlap ONECUT-motif-CRs in the *OC1-up, OC2-up* and *OC3-up* CR sets with ONECUT-motif-CRs in the *iN-Fib-up* CR set. Relationship between the ONECUT motif score threshold and the percentage of overlap. (**E**) Odds ratios for the overlap of CRs differentially accessible following OC1/2/3 overexpression with iNeuron CRs and CRs accessible in ENCODE/ROADMAP cell types. *ER-OC1-up, ER-OC2-up, ER-OC3-up, ER-OC1-down, ER-OC2-down and ER-OC3-down* are the differentially accessible CR sets filtered for CRs also present in the DNase union (see Materials and Methods). The density plot is across all ENCODE/ROADMAP DNase samples. Circles indicate the means with standard deviations for odds ratios in the selected groups of DNase samples; stars indicate the odds ratios for individual biological replicates from this study. (**F**) Lineage specificity of differentially accessible CRs. Lineage-specific OCRs (LSOCRS) were defined as specific to either iNeurons or an ENCODE/ROADMAP lineage (i.e., only found in samples within that lineage but not outside it, see Methods). Shown are the percentage of LSOCRs across lineages. For each differential CR set, the percentage of LSOCRs across lineages add up to 100%. Rows are ordered on the percentage of LSOCRs for the OC1-up CR set.

First, we characterized overall similarity of the genome-wide chromatin accessibility using Pearson correlations of the quantile normalized log-transformed ATAC-seq fragment counts (FPKMs). We found that the OC1/2/3 conditions were more similar to each other and to fibroblasts than to iNeurons (Figure 5A). Thus, with linear correlation of chromatin accessibility as a measure of overall similarity, the OC1/2/3 conditions still differ substantially from iNeurons. In a principal component analysis, OC1/2/3 overexpression conditions also clustered separately from iNeurons (Supplementary Figure S10C).

Second, we compared the chromatin accessibility changes induced by OC1/2/3 overexpression, denoted by Δ(OC1/2/3+Bclxl,Bclxl), to the difference in chromatin accessibility between iNeurons and fibroblasts, denoted by Δ(iNeuron,Fibroblast). For all three ONECUT factors, overexpression resulted in enhanced chromatin accessibility at CRs more accessible in iNeurons than in fibroblasts (see upper right quadrants defined by Δ(iNeuron,Fibroblast) > 1 and Δ(OC1/2/3+Bclxl,Bclxl) > 1 in Figure 5B). However, chromatin accessibility was also enhanced at CRs that were not more accessible in iNeurons than in fibroblasts (see positive horizontal axis defined by Δ(iNeuron,Fibroblast) ≈ 0 and Δ(OC3+Bclxl,Bclxl) > 1 in Figure 5B). When we restricted this analysis to the subset of ONECUT-motif-CRs (Figure 5C), the picture is somewhat different. Since most ONECUT-motif-CRs from the ATAC union set had low accessibility in fibroblasts, and the ONECUT motif was positively correlated with chromatin differences between iNeurons and fibroblasts, one would expect most of the changes in accessibility to fall into the upper right quadrant (Figure 5C). This is indeed what we observed: the majority of ONECUT-motif-CRs had higher accessibility in iNeurons than in fibroblasts, and many ONECUT-motif-CRs had higher accessibility following overexpression of OC1/2/3. However, for each ONECUT factor, there was a subset a ONECUT-motif-CRs that were not differentially accessible between iNeurons and fibroblasts, but which nonetheless gained accessibility following overexpression (Figure 5C).

Third, we quantified overlap by intersecting the differential CR sets for the ONECUT factors (*OC1-up, OC2-up, OC3-up* and *OC1-down, OC2-down, OC3-down*) with the set of CRs differentially accessible between iNeurons and fibroblasts (*iN-fib-up, iN-fib-down*). We found substantial overlap between CRs upregulated by the ONECUT factors and CRs upregulated in iNeurons relative to fibroblasts (Figures 3F and 4F), and similarly for downregulated CRs (Figures 3G and 4G). ONECUT-motif-CRs upregulated by OC1/2/3 were more likely to overlap with an iNeuron CR when the motif score was higher and the motif prediction is expected to be more accurate (Figure 5D). However, many CRs that became accessible following OC overexpression were not shared with iNeurons (Figure 3F).

Fourth, we investigated whether ONECUT factors preferentially enhanced the accessibility at CRs accessible in iNeurons relative to CRs accessible in other cell types (see Materials and Methods). As there is currently no large systematic characterization of chromatin accessibility in a wide range of tissues by ATAC-seq, we created a union (‘DNase union’) of OCRs identified by DNase-seq of 53 different tissues and cell types in the ENCODE and ROADMAP projects (28,29). To reduce a potential bias towards iNeurons, we quantified both DNase-seq and ATAC-seq fragment counts on the DNase union set so that iNeuron CRs identified only by ATAC-seq were excluded (see Methods). We then used DESeq2 to identify up- and downregulated CRs (defined respectively as *ER-OC1-Up, ER-OC2-Up and ER-OC3-Up, ER-OC1-Down, ER-OC2-Down and ER-OC3-Down)*. We found that CRs differentially more accessible following OC1/2/3 overexpression (*ER-OC1-up, ER-OC2-up* and *ER-OC3-up*) were more likely to overlap with CRs accessible in iNeurons than with CRs accessible in ROADMAP DNase-seq samples, including neuronal or brain samples from ROADMAP (Figure 5E, Supplementary Table S4). However, the odds-ratios were moderate, likely because many CRs that gained accessibility after OC1/2/3 overexpression were not accessible in any iNeuron replicate (resp. 71%, 75% and 66%).

Last, to determine whether CRs that gain accessibility (*OC1-up, OC2-up* and *OC3-up*) are associated with a specific cell lineage, we focused on lineage specific OCRs (LSOCRs). We grouped ENCODE/ROADMAP DNase-seq samples on cell lineage (according to ENCODE/ROADMAP annotations) and added iNeurons as a separate lineage. We then defined LSOCRs as CRs that are accessible in only one lineage (see Methods). LSOCRs that gained accessibility following OC1/2/3 overexpression were not restricted to a single lineage (Figure 5F, Supplementary Figure S6B). Chromatin accessibility was increased most frequently at LSOCRs from iNeurons (Figure 5F, Supplementary Figure S10D), although we cannot rule out that this may be partly explained by sequence bias of ATAC-seq versus DNAse-seq.

Taken together, these analyses support the notion that in terms of chromatin accessibility, overexpression of OC1/2/3 did not increase overall similarity with iNeurons, despite the overlap of induced CRs with iNeuron OCRs.

### OC1/2/3 overexpression induces expression of genes in neuronal and non-neuronal pathways

**Figure 6.**
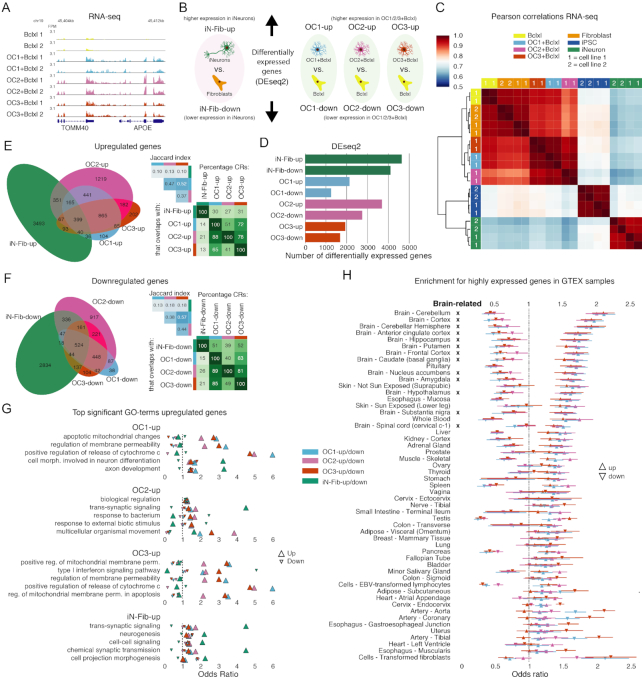
Gene expression changes induced by overexpression of ONECUT factors in fibroblasts. (**A**) Representative example of RNA-seq data on day 2 after induction of ONECUT transgene expression. Shown is the region containing the APOE gene, which is differentially upregulated by OC1, OC2 and OC3. FPM, fragments per million. (**B**) Definition of sets of differentially expressed genes, as determined by DESeq2 using GENCODE 27 gene definitions and RNA-seq read counts. (**C**) Hierarchically clustered heatmap of Pearson correlations of the quantile normalized log2(FPKM+1) RNA-seq fragment counts. Row and column colors indicate cell type, numbers inside the row and column colors indicate cell line. (**D**) Number of genes in the differentially expressed gene sets. (**E**) Venn diagram, Jaccard indices and overlap percentages for the overlap of genes upregulated following OC1/2/3 overexpression and genes upregulated in iNeurons relative to fibroblasts (top row in panel B). Venn diagram areas are approximately proportional to the number of CRs. (**F**) Venn diagram, Jaccard indices and overlap percentages for the overlap of genes downregulated following OC1/2/3 overexpression and genes downregulated in iNeurons relative to fibroblasts (top row in panel B). Venn diagram areas are approximately proportional to the number of CRs. (**G**) GO-terms associated with differentially upregulated genes. Similar GO-terms were discarded based of information content in the GO-term graph (see Methods). For the five most significant GO-terms associated with respectively *OC1-up, OC2-up, OC3-up* and *iN-Fib-up*, the odds ratios are plotted for all differentially expressed gene sets (panel B). (**H**) Odds ratios for the overlap of differentially expressed gene sets (*OC1-up, OC2-up, OC3-up, OC1-down, OC2-down,OC3-down)* with genes highly expressed (log2(FPKM+1) > 6) in 8555 samples from the GTEX project (31). Plotted as triangles are the mean odd ratio and standard deviation for GTEX samples of the same tissue or cell type. The rows are ordered by the mean odds ratio for OC1. Brain-related GTEX samples are marked with an X.

We profiled gene expression (RNA-seq) in fibroblasts overexpressing either Bclxl (control), OC1 and Bclxl, OC2 and Bclxl or OC3 and Bclxl at the same time point as the ATAC-seq data (2 days after induction) (Figures 2A and 6A). We used DESeq2 (22) to identify genes that are differentially expressed after OC1/2/3 overexpression (Figure 6B).

We characterized overall similarity of the gene expression using Pearson correlation coefficients of the quantile normalized log-transformed gene expression fragment counts (FPKMs). After two days of OC1/2/3 overexpression, gene expression was more similar to that in fibroblasts than to that of iNeurons (Figure 6C). The overexpression of OC1/2/3 resulted in respectively 2135, 3689 and 1933 significantly upregulated genes and respectively 1248, 2741 and 1681 significantly downregulated genes (Figure 6D). There was a large degree of overlap of differentially expressed genes between OC1, OC2 and OC3 (Figure 6E and F). The majority of genes that were differentially expressed between iNeurons and fibroblasts were not upregulated or downregulated by overexpression of OC1/2/3 (Figure 6E and F).

To characterize the biological function of the differentially expressed genes, we performed pathway analysis using GOstats (30). Genes upregulated by OC1/2/3 (*OC1-up, OC2-up* and *OC3-up*) were enriched for pathways involving neuronal function or development (trans-synaptic signaling, axon development and cell morphogenesis involved in neuron differentiation) (Figure 6G, Supplementary Figure S11A, Supplementary Table S5), but also for non-neuronal pathways such as apoptotic mitochondrial changes and type I interferon signaling (Figure 6G, Supplementary Table S5). The neuronal pathway trans-synaptic signaling was the GO-term most significantly associated with *iN-Fib-up* genes and was also associated with *OC1-up, OC2-up* and *OC3-up* genes (Figure 6G). Overexpression of OC1/2/3 in some cases resulted in the simultaneous up- and down-regulation of genes in the same neuronal pathway: for instance, the axon development pathway was enriched in both *OC1-up* and *OC1-down* genes (Figure 6G, Supplementary Figure S11A). In contrast, only *iN-Fib-up* but not *iN-Fib-down* genes were enriched in this pathway (Figure 6G, Supplementary Figure S11A) Pathways associated with genes downregulated by OC1/2/3 (*OC1-down, OC2-down* and *OC3-down*) were highly similar to those associated with genes downregulated in iNeurons relative to fibroblast (*iN-Fib-down*) and included cardiovascular and cell motility pathways (Supplementary Figure S11B, Supplementary Table S6).

To investigate whether OC1/2/3 overexpression induced the expression of genes expressed in a specific tissue or cell type, we determined for >8000 GTEX (31) RNA-seq samples which genes are expressed and which genes are highly expressed (Supplementary Figure S11C). We intersected these genes with the differentially expressed genes (Figure 6B) and determined the odds ratio of: (i) the odds that a gene that was differentially expressed following OC1/2/3 overexpression was also (highly) expressed in a specific GTEX sample, and (ii) the odds that a random gene was (highly) expressed in that GTEX sample. The overlap with the genes expressed in the GTEX samples suggested that upregulated genes are often expressed in brain tissue, compared to other tissues, but differences in odds ratios between tissues were small (Supplementary Figure S11D). When we restricted the analysis to highly expressed genes, these differences became larger (Figure 6H). Of 53 different tissues, the 13 brain tissues were within the top 18 tissues with highest odds ratio for overlap with genes upregulated by OC1 (Figure 6H). In addition, relatively few genes highly expressed in brain were downregulated (Figure 6H). The pattern for OC2 and OC3 was similar to that of OC1 (Figure 6H).

Finally, we asked whether there is a relationship between the changes in gene expression and changes in chromatin accessibility induced by OC1/2/3 overexpression. We investigated whether there is an enrichment of differential CRs near differential genes compared to other genes. For OC1 and OC2 we found that the genomic regions of upregulated genes were enriched for *OC1/2-up* CRs and the genomic regions of downregulated genes were enriched for *OC1/2-down* CRs (Supplementary Figure S12a,b,c). For OC3, the transcription start sites (TSSs) of differential genes (both up- and downregulated) were depleted for *OC3-up* CRs and enriched for *OC3-down* CRs (Supplementary Figure S12a). More distally, *OC3-down* genes were depleted for *OC3-down* CRs (Supplementary Figure S12B and C).

### OC1/2/3-up and iN-Fib-up ONECUT-motif-CRs are associated with different transcription factor motifs and histone modifications

We found above that not all *iN-Fib-up* ONECUT-motif-CRs gained accessibility after OC1/2/3 overexpression (Figures 4F and 5D) and identified *OC1-up, OC2-up* and *OC3-up* ONECUT-motif-CRs that were not more accessible in iNeurons than in fibroblasts (Figure 4F). To identify potential mechanisms or co-factors that influence the effect of OC1/2/3 overexpression, we asked if specific histone modifications or additional TF motifs were associated with ONECUT-motif-CR subsets not shared between iNeurons and OC1/2/3 overexpression conditions.

**Figure 7.**
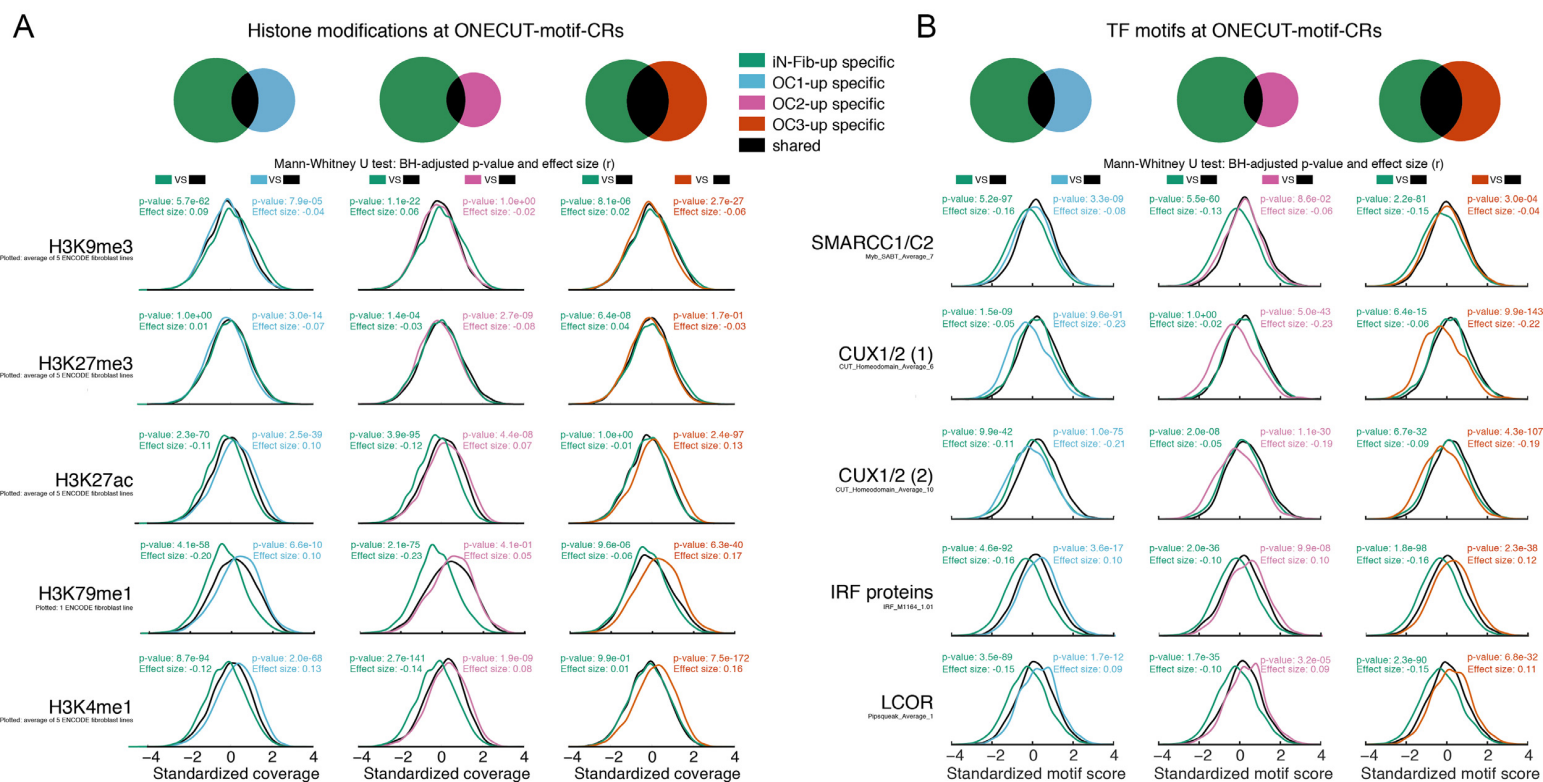
Analysis of histone modifications and TF motif differences between ONECUT overexpression and iNeurons. (**A**) Histone modification comparison at differentially more accessible ONECUT-motif-CRs. The figure shows the histone modification coverage distributions in ENCODE/ROADMAP fibroblast samples at ONECUT-motif-CRs respectively specific to *iN-Fib-up* CRs (green), specific to *OC1/2/3-up (blue/pink/orange)* and shared between *iN-Fib-up* and *OC1/2/3-up (black)*. Histone modification coverage was quantified at the ATAC Union CRs and standardized (mean = 0, standard deviation = 1) using all ATAC Union CRs. To quantify the shift in distribution across specific CRs relative to the shared CRs, we used a Mann-Whitney U test (see Methods). Shown are the corresponding Benjamini-Hochberg adjusted *P*-values and effect sizes. Distributions are shown for H3K9me3, H3K29me3, H3K27ac and two histone modification with the largest observed significant effect sizes (BH-adjusted *P*-value < 0.05): H3K29me1 and H3K4me1. (**B**) TF motif comparison at differentially more accessible ONECUT-motif-CRs. The figure shows TF motif score distributions at ONECUT-motif-CRs respectively specific to *iN-Fib-up* CRs (green), specific to *OC1/2/3-up (blue/pick/orange)* and shared between *iN-Fib-up* and *OC1/2/3-up (black)*. Motif scores were quantified at the ATAC Union CRs and standardized (mean = 0, standard deviation = 1) using all ATAC Union CRs. To quantify the shift in motif score distribution across specific CRs relative to the shared CRs, we used a Mann-Whitney U test (see Methods). Distributions are shown for the five motifs with the largest significant effect sizes (BH-adjusted *P*-value < 0.05).

We determined the coverage for 25 different histone modifications, each one assayed in up to five ENCODE/ROADMAP fibroblast samples (E017, E055, E056, E126 and E128; Supplementary Table S7) at ONECUT-motif-CRs that were either shared between or specific to *iN-Fib-up* CRs and respectively *OC1-up, OC2-up* or *OC3-up* CRs. We used the Mann-Whitney-U test to compare the coverage distribution of each histone modification at these different ONECUT-motif-CR sets. We found evidence for differences between these subsets for H3K79me1 (active transcription) and H3K4me1 (associated with enhancers) (Figure 7A, Supplementary Figures S13A and B)

We calculated motif scores for 580 clustered TF motifs based on the cis-bp database (26) using GimmeMotifs (25) at the same ONECUT-motif-CRs. We found that *iN-Fib-up-*specific ONECUT-motif-CRs were depleted for motifs associated with SMARCC1/SMARCC2, IRF proteins, SP1–9, ZBTB7B, FOX proteins and a motif associated with both NR1D1/NR1D2 and RORA/RORB/RORC, compared to those shared with *OC1-up, OC2-up* and *OC3-up* (Figure 7B, Supplementary Figure S13C). *OC1-up, OC2-up* and *OC3-up*-specific ONECUT-motif-CRs were enriched for motifs associated with IRF protein and LCOR and depleted for motifs associated with CUX1/CUX2, compared to those shared with *iN-Fib-up*. (Figure 7B, Supplementary Figure S13D).

## DISCUSSION

In this study, we found that the ONECUT TF motif was strongly associated with differential chromatin accessibility between iNeurons (obtained by overexpression of Neurog2 in human iPSCs) and fibroblasts. All three ONECUT factors induced a neuron-like morphology and expression of neuronal genes within two days after overexpression in fibroblasts. We observed widespread remodeling of chromatin accessibility; in particular, we found that chromatin regions that contained the ONECUT motif were in- or lowly accessible in fibroblasts and became accessible after the overexpression of a ONECUT factor. There was substantial overlap with iNeurons, still, many regions that gained accessibility following ONECUT overexpression were not accessible in iNeurons. Genome-wide, chromatin accessibility and gene expression did not become more similar to that of iNeurons.

TFs that can bind and open nucleosomal DNA at their cognate binding sites are known as pioneer factors (32). Examples are Ascl1 (17), SOX2 (33) and PAX7 (34). It was previously shown that ONECUT1 binds accessible chromatin in iPSC-derived motor neurons and that 75% of the 142,000 occurrences of the 8 bp Onecut consensus motif in the mouse genome are occupied by Onecut1 (35). Here, we found that the ONECUT sequence motif was strongly associated with chromatin regions that gained accessibility following OC1/2/3 overexpression (Figure 4B and D). Many ONECUT-motif-CRs with low accessibility in fibroblasts became more accessible (Figure 4A and H) and the ATAC-seq data strongly suggest that TFs were bound at the ONECUT motif in CRs that gained accessibility (Figure 4H and I). In addition, no motifs clearly distinct from the ONECUT motif were enriched in ONECUT-motif-CRs that gained accessibility following OC1/2/3 overexpression (Figure 4H, Supplementary Figure S10A). From this we cannot fully conclude that ONECUT TFs function as pioneer factor in this context. Nonetheless, our findings support a model in which overexpressed ONECUT factors bind to the DNA in previously poorly accessible chromatin that becomes more accessible. This property of ONECUT TFs would be consistent with that of pioneer factors. Whether ONECUT TFs function as pioneer factors when overexpressed in fibroblasts remains to be clarified and should be verified experimentally, for example by nucleosome reconstitution assays (36).

ONECUT TFs were previously found to bind together with cofactors to the DNA to regulate gene expression (35,37). Our results show that a substantial number of ONECUT-motif-CRs that were accessible in iNeurons did not gain accessibility following OC1/2/3 overexpression (Figure 4F) and that regions accessible in iNeurons with a high ONECUT motif score are more likely to gain accessibility following OC1/2/3 overexpression than those with a lower motif score (Figure 5D). Thus, the question remains whether there are cofactors in iNeurons, likely not present in fibroblasts, that facilitate the opening of ONECUT-motif-CRs in iNeurons. Our finding that histone modifications and specific transcripton factor motifs were associated with *iN-Fib-up* ONECUT-motif-CRs that did not gain accessibility after OC1/2/3 overexpression, supports the involvement of co-factors (Figure 7A and B). For instance, CUX1 and/or CUX2 may cooperate with OC1/2/3 to enhance accessibility of a specific set of ONECUT-motif-CRs in iNeurons as we found their associated motif to be enriched in ONECUT-motif-CRs that are accessible in iNeurons but did not gain accessibility following ONECUT overexpression (Figure 7B, Supplementary Figure S13E). Conversely, we found that the DNA binding motif associated with LCOR is enriched at ONECUT-motif-CRs that gain accessibility following OC1/2/3 overexpression but are not more accessible in iNeurons than in fibroblasts (Figure 7B). LCOR is a transcriptional corepressor (38) and is highly expressed in iNeurons compared to fibroblast and not upregulated by OC1/2/3 overexpression (Supplementary Figure S13E). Although speculative, it is possible that in iNeurons LCOR prevent OC1/2/3 from enhancing accessibility at ONECUT-motif-CRs that should not gain accessibility and that lack of LCOR expression allows OC1/2/3 to enhance accessibility at these ONECUT-motif-CRs. Whether CUX1/2 and LCOR indeed cooperate with the ONECUT TFs to regulate the chromatin accessibility in iNeurons requires further investigation.

A separate but related issue are changes at CRs that do not contain the ONECUT-motif. Overexpression of OC1/2/3 resulted in increased accessibility at many CRs that are not accessible in iNeurons, and vice versa, reduced accessibility at CRs that are accessible in iNeurons (Figure 5B). A key question is whether these are direct or indirect consequences of the ONECUT overexpression. CRs without the ONECUT motif that gained accessibility following OC1 or OC2 overexpression were enriched for a motif associated with PBX TFs (Figure 4I, Supplementary Figure S10A). In future studies, ChIP-seq studies of ONECUT binding may be used to determine whether ONECUT factors can bind in CRs without the canonical ONECUT motif, or whether chromatin changes at these CRs are an indirect consequence of ONECUT overexpression. In two previous studies on embryonic stem cell motor neuron differentiation ONECUT TFs cooperated with other TFs to activate gene expression (35,37). A more detailed understanding of the chromatin context, co-factors and the timing of ONECUT TF expression in iNeuron differentiation may help to resolve these questions.

The effects of OC1, OC2 and OC3 overexpression in fibroblasts were similar at multiple levels. OC1/2/3 all induced neuron-like morphology (Figure 2B) and upregulated genes expressed in brain-related tissues (Figure 6H). All three ONECUT factors preferentially enhanced the accessibility of ONECUT-motif-CRs (Figure 4B, D) and CRs accessible in iNeurons (Figure 5E, F). However, there were also clear differences, illustrated by the degree of overlap between induced CRs (Figure 3F). We cannot rule out that the differences that were observed between OC1/2/3 are due to differences in the level of overexpression and virus titer. Furthermore, we observed that the influence of the MACS2 peak calling threshold on the number of identified OCRs was much larger for OC3 than for OC1 and OC2 (Supplementary Figure S14). This may also affect the analyses where we investigated the intersection of differentially accessible CRs (Figures 3F,G, 4F,G). Previous studies have shown that in mouse liver and retina development, OC1 and OC2 have partially redundant functions (39,40), and that in mouse dopaminergic neuron development there is some redundancy between OC1 and OC2 and/or OC3 (41). Our data are consistent with these findings and suggest that this redundancy may be partially present at the chromatin level. The structure and DNA binding mechanism of OC1 has previously been investigated in detail (42). Further studies on the structural differences between OC1, OC2, OC3 might help to better understand the similarities and differences in the chromatin remodeling capacities of OC1, OC2 and OC3.

There are a number of limitations to this study. First, we did not discriminate between the DNA binding motif of OC1, OC2 and OC3. Second, the apoptosis induced by OC1/2/3 overexpression has likely affected both the ATAC-seq and RNA-seq data. Genes associated with apoptosis were upregulated (Figure 6G) and the motif of the apoptosis associated IRF proteins was enriched in the CRs that gain accessibility after OC1/2/3 overexpression (Supplementary Figure S8C–E). Third, OC1/2/3 overexpression resulted in a heterogeneous cell population with neuron-like cells, cells that retained a fibroblast-like morphology and cells undergoing apoptosis. Given the limitations of bulk ATAC-seq and RNA-seq assays, we could not discriminate between these subpopulations in the computational analyses. In future studies, single-cell ATAC-seq (43) may be used to resolve this heterogeneity. Last, we did not discriminate between OCRs that contribute to gene expression and those that do not (e.g. by including H3K27ac data to identify active enhancers).

We performed the validation experiments in fibroblast medium rather than neuronal medium so that the chromatin remodeling effects of ONECUT overexpression would not be confounded by potential chromatin changes induced by growth factors or HDAC inhibitors (e.g. as in (15)) that may be present in the neuronal differentiation medium. To investigate the full potential of OC1/2/3 for direct neuronal reprogramming, it will likely be necessary to perform these experiments in neuronal differentiation medium and co-culture the cell with supporting astrocytes, similar to the hiPSC-iNeuron differentiation protocol. In light of the results of the histone modification analysis (Figure 7A, Supplementary Figure S13A, B), it will be of interest to consider small compounds that modify chromatin state as these have previously been effective in direct neuronal reprogramming (8,9,16). Overexpression of CUX1, CUX2 and LCOR could potentially more specifically recreate the chromatin accessibility profile of iNeurons. Although it remains to be investigated to what extent iNeuron chromatin accessibility represents any *in vivo* neuronal chromatin state, the rapid chromatin remodeling and induction of neuronal characteristics suggest that the ONECUT factors may be a promising avenue to develop new direct neuronal reprogramming protocols.

## DATA AVAILABILITY

The sequence data are available from GEO under accession number GSE120131.

## Supplementary Material

gkz273_Supplemental_File

## Notes

Present address: Cornelis A. Albers. Euretos BV, Utrecht, The Netherlands
