# Supplementary material for: ONECUT transcription factors induce neuronal characteristics and remodel chromatin accessibility
Source: Nucleic Acids Res. 2019 May 3;47(11):5587–602. doi: 10.1093/nar/gkz273 (PMC6582315; doi:10.1093/nar/gkz273)
Supplement: gkz273_Supplemental_File [file gkz273_supplemental_file.pdf]

Supplementary information

ONECUT transcription factors induce neuronal characteristics and remodel chromatin accessibility

Jori van der Raadt[1,2], Sebastianus H.C. van Gestel[1,2], Nael Nadif Kasri[1,3], Cornelis A. Albers[1,2]

[1]Department of Human Genetics, Donders Centre for Neuroscience, Radboud University Medical Center, Nijmegen, The Netherlands

[2]Department of Molecular Developmental Biology, Radboud institute for Molecular Life Sciences, Radboud University, Nijmegen, The Netherlands

[3]Department of Cognitive Neuroscience, Donders Centre for Neuroscience, Radboud University Medical Center, Nijmegen, The Netherlands

Correspondence to: Jori van der Raadt, jorivdr@gmail.com

Cornelis A. Albers, kees.albers@radboudumc.nl

Table S1. Details of cell lines used. The fibroblast and iPSC cell lines originate from the Radboud University Medical Center Human Genetics biobank, Nijmegen, The Netherlands.

Name	Name in biobank	sex	age	Lentiviral transgenes	Origin	Used for
Fibroblast line 1	CL15-00023	female	29	rtTA		ATAC/RNA fibroblasts, ATAC/RNA/morphology validations
Fibroblast line 2	CL15-00024	male	9	rtTA		ATAC/RNA fibroblasts, morphology validations
Fibroblast line 3	CL16-00044	female	44	rtTA, Neurog2		Morphology validations
iPSC line 1	IPS15-00030			OSKM, rtTA, Neurog2	Fibroblast line 1	ATAC/RNA iPSCs
iPSC line 2	IPS15-00024			OSKM, rtTA, Neurog2	Fibroblast line 2	ATAC/RNA iPSCs
iNeuron line 1				OSKM, rtTA, Neurog2	iPSC line 1	ATAC/RNA iNeurons
iNeuron line 2				OSKM, rtTA, Neurog2	iPSC line 1	ATAC/RNA iNeurons

Table S2. Lentiviral transfer vectors.

Name	Transgene	Accession	Vector description	Antibiotic resistance	Source
rtTA	rtTA	-	pLVX-EF1α-rtTA-IRES-G418R	G418	Oliver Brüstle
Ngn2	Mouse Neurog2	CCDS17825.1	pLVX-TRE-mNeurog2-PGK-puroR	Puromycin	Oliver Brüstle
Bclxl	Human BCL2L1	NM_138578	pCDH-puro-Bclxl	Puromycin	Addgene #46972
ONECUT1	Human ONECUT1	NM_004498.2	pLV-TRE-hONECUT-PGK-Bsd	Blasticidin	Cyagen VB160805-1096fer
ONECUT2	Human ONECUT2	Vectorbuilder ORF022212	pLV-TRE-hONECUT2-PGK-Bsd	Blasticidin	Cyagen VB160804-1074sdt
ONECUT3	Human ONECUT3	NM_001080488.1	pLV-TRE-hONECUT3-PGK-Bsd	Blasticidin	Cyagen VB160802-1049kde

Table S3. Mapped reads for ATAC-seq and RNA-seq data

ATAC-seq

Condition	Cell line	Replicate	Mapped reads after filtering
Fibroblast	Fibroblast line 1	1	30,998,018
Fibroblast	Fibroblast line 1	2	23,275,133
Fibroblast	Fibroblast line 2	1	15,359,048
Fibroblast	Fibroblast line 2	2	22,236,076
iPSC	iPSC line 1	1	18,125,553
iPSC	iPSC line 1	2	16,350,555
iPSC	iPSC line 2	1	24,748,036
iPSC	iPSC line 2	2	28,419,033
iNeuron	iNeuron line 1	1	17,279,426
iNeuron	iNeuron line 1	2	10,052,596
iNeuron	iNeuron line 2	1	22,694,340
iNeuron	iNeuron line 2	2	137,233,084
Bclxl	Fibroblast line 1	1	14,915,892
Bclxl	Fibroblast line 1	2	12,003,541
OC1+Bclxl	Fibroblast line 1	1	23,702,029
OC1+Bclxl	Fibroblast line 1	2	18,155,089
OC2+Bclxl	Fibroblast line 1	1	21,072,955
OC2+Bclxl	Fibroblast line 1	2	39,173,763
OC3+Bclxl	Fibroblast line 1	1	22,692,776
OC3+Bclxl	Fibroblast line 1	2	15,446,908

RNA-seq

Condition	Cell line	Replicate	Mapped reads after filtering
Fibroblast	Fibroblast line 1	1	44,483,036
Fibroblast	Fibroblast line 1	2	51,015,494
Fibroblast	Fibroblast line 2	1	31,409,042
Fibroblast	Fibroblast line 2	2	46,274,810
iPSC	iPSC line 1	1	39,733,014
iPSC	iPSC line 1	2	45,655,986
iPSC	iPSC line 2	1	26,594,872
iPSC	iPSC line 2	2	20,574,928
iNeuron	iNeuron line 1	1	11,077,548
iNeuron	iNeuron line 1	2	6,945,160
iNeuron	iNeuron line 2	1	16,514,506
iNeuron	iNeuron line 2	2	11,252,892
Bclxl	Fibroblast line 1	1	27,382,802
Bclxl	Fibroblast line 1	2	64,325,334
OC1+Bclxl	Fibroblast line 1	1	19,577,134
OC1+Bclxl	Fibroblast line 1	2	51,424,622
OC2+Bclxl	Fibroblast line 1	1	24,926,284
OC2+Bclxl	Fibroblast line 1	2	23,227,206
OC3+Bclxl	Fibroblast line 1	1	26,327,440
OC3+Bclxl	Fibroblast line 1	2	50,046,594

Table S4. Odds ratios ENCODE/ROADMAP samples for overlap with differential CRs

Top 5 highest (1-5) and lowest (49-53) odds ratios ENCODE/ROADMAP samples for overlap with CRs OC1/2/3 up

Rank	**OC1 up**	**OC2 up**	**OC3 up**
1	NHEK-Epidermal Keratinocyte Primary Cells	NHEK-Epidermal Keratinocyte Primary Cells	GM12878 Lymphoblastoid Cells
2	GM12878 Lymphoblastoid Cells	GM12878 Lymphoblastoid Cells	NHEK-Epidermal Keratinocyte Primary Cells
3	K562 Leukemia Cells	K562 Leukemia Cells	K562 Leukemia Cells
4	Small Intestine	Primary T cells from peripheral blood	Small Intestine
5	Primary T cells from peripheral blood	Primary T cells from cord blood	Primary T cells from peripheral blood
49	iPS DF 6.9 Cells	Fetal Muscle Trunk	Fetal Kidney
50	Fetal Lung	Fetal Lung	HSMM derived Skeletal Muscle Myotubes Cells
51	H9 Cells	H1 Derived Neuronal Progenitor Cultured Cells	Foreskin Fibroblast Primary Cells skin02
52	H1 Derived Neuronal Progenitor Cultured Cells	Fetal Kidney	Foreskin Fibroblast Primary Cells skin01
53	Fetal Kidney	Fetal Muscle Leg	NHDF-Ad Adult Dermal Fibroblast Primary Cells

Top 5 highest (1-5) and lowest (49-53) odds ratios ENCODE/ROADMAP samples for overlap with CRs OC1/2/3 down

Rank	**OC1 down**	**OC2 down**	**OC3 down**
1	NHDF-Ad Adult Dermal Fibroblast Primary Cells	NHDF-Ad Adult Dermal Fibroblast Primary Cells	NHDF-Ad Adult Dermal Fibroblast Primary Cells
2	HSMM Skeletal Muscle Myoblasts Cells	HSMM Skeletal Muscle Myoblasts Cells	Foreskin Fibroblast Primary Cells skin02
3	Foreskin Fibroblast Primary Cells skin02	Foreskin Fibroblast Primary Cells skin02	HSMM Skeletal Muscle Myoblasts Cells
4	NHLF Lung Fibroblast Primary Cells	NHLF Lung Fibroblast Primary Cells	Foreskin Fibroblast Primary Cells skin01
5	NH-A Astrocytes Primary Cells	NH-A Astrocytes Primary Cells	NHLF Lung Fibroblast Primary Cells
49	H9 Cells	Primary NK cells from peripheral blood	Fetal Intestine Small
50	Fetal Intestine Small	Fetal Intestine Small	Fetal Intestine Large
51	Primary T cells from cord blood	Primary T cells from cord blood	Primary T cells from cord blood
52	Primary T cells from peripheral blood	Primary T cells from peripheral blood	Primary B cells from peripheral blood
53	Primary B cells from peripheral blood	Primary B cells from peripheral blood	Primary T cells from peripheral blood

Table S5. GO-terms associated with upregulated genes

GO-terms associated to genes upregulated by ONECUT1

	ID	GO-term	p-value (FDR-adjusted)	Odds ratio	Associated differential genes	Total associated genes
1	GO:0008637	apoptotic mitochondrial changes	3.14E-04	2.88	37	109
2	GO:0090559	regulation of membrane permeability	8.66E-04	3.40	25	66
3	GO:0090200	positive regulation of release of cytochrome c from mitochondria	8.66E-04	5.99	14	27
4	GO:0048667	cell morphogenesis involved in neuron differentiation	8.66E-04	1.68	101	441
5	GO:0061564	axon development	8.66E-04	1.72	91	389
6	GO:0023052	signaling	9.00E-04	1.31	386	2200
7	GO:2001233	regulation of apoptotic signaling pathway	9.25E-04	1.74	80	339
8	GO:0010646	regulation of cell communication	9.25E-04	1.27	462	2577
9	GO:0035794	positive regulation of mitochondrial membrane permeability	9.25E-04	3.60	20	51
10	GO:0023051	regulation of signaling	9.25E-04	1.27	470	2625

GO-terms associated to genes upregulated by ONECUT2

	ID	GO-term	p-value (FDR-adjusted)	Odds ratio	Associated differential genes	Total associated genes
1	GO:0065007	biological regulation	5.74E-06	1.27	2400	9542
2	GO:0099537	trans-synaptic signaling	6.16E-06	1.72	171	496
3	GO:0009617	response to bacterium	6.59E-04	1.67	125	369
4	GO:0043207	response to external biotic stimulus	6.59E-04	1.50	191	607
5	GO:0050879	multicellular organismal movement	9.00E-04	3.87	24	44
6	GO:0007267	cell-cell signaling	1.01E-03	1.38	273	927
7	GO:0090200	positive regulation of release of cytochrome c from mitochondria	1.10E-03	4.97	17	28
8	GO:0003015	heart process	1.10E-03	1.80	81	227
9	GO:0048731	system development	1.10E-03	1.21	828	3143
10	GO:0051591	response to cAMP	1.10E-03	2.54	37	84

GO-terms associated to genes upregulated by ONECUT3

	ID	GO-term	p-value (FDR-adjusted)	Odds ratio	Associated differential genes	Total associated genes
1	GO:0035794	positive regulation of mitochondrial membrane permeability	5.89E-03	3.45	18	51
2	GO:0060337	type I interferon signaling pathway	5.89E-03	3.02	21	65
3	GO:0090559	regulation of membrane permeability	5.89E-03	3.02	21	65
4	GO:0090200	positive regulation of release of cytochrome c from mitochondria	5.89E-03	4.74	12	28
5	GO:1902110	positive regulation of mitochondrial membrane permeability in apoptosis	5.89E-03	3.36	17	49
6	GO:0043207	response to external biotic stimulus	5.89E-03	1.50	110	579
7	GO:0034340	response to type I interferon	5.89E-03	2.83	21	68
8	GO:0035556	intracellular signal transduction	5.89E-03	1.26	371	2306
9	GO:0042026	protein refolding	7.73E-03	5.26	10	22
10	GO:0012501	programmed cell death	2.67E-02	1.45	94	516

GO-terms associated to genes higher expressed in iNeurons than in fibroblasts

	ID	GO-term	p-value (FDR-adjusted)	Odds ratio	Associated differential genes	Total associated genes
1	GO:0099537	trans-synaptic signaling	1.71E-48	4.52	302	529
2	GO:0022008	neurogenesis	1.12E-17	2.19	421	1085
3	GO:0007267	cell-cell signaling	1.25E-16	3.02	219	467
4	GO:0007268	chemical synaptic transmission	4.60E-12	4.25	120	216
5	GO:0048858	cell projection morphogenesis	6.08E-12	2.26	303	749
6	GO:0007156	homophilic cell adhesion via plasma membrane adhesion molecules	1.08E-09	5.16	91	148
7	GO:0000904	cell morphogenesis involved in differentiation	8.29E-09	2.48	214	500
8	GO:0030182	neuron differentiation	1.28E-06	2.28	222	557
9	GO:0006813	potassium ion transport	4.48E-05	3.64	98	185
10	GO:0034765	regulation of ion transmembrane transport	1.52E-04	3.17	112	227

Table S6. GO-terms associated with downregulated genes

GO-terms associated to genes downregulated by ONECUT1

	ID	GO-term	p-value (FDR-adjusted)	Odds ratio	Associated differential genes	Total associated genes
1	GO:2000145	regulation of cell motility	1.85E-11	2.41	113	629
2	GO:0051674	localization of cell	1.85E-11	2.02	172	1127
3	GO:0072358	cardiovascular system development	3.03E-08	2.24	93	544
4	GO:0001568	blood vessel development	4.39E-07	2.17	85	510
5	GO:0090132	epithelium migration	1.06E-06	2.93	45	209
6	GO:0001655	urogenital system development	3.42E-06	2.50	54	285
7	GO:0051271	negative regulation of cellular component movement	4.28E-06	2.73	45	221
8	GO:0044707	single-multicellular organism process	4.28E-06	1.41	440	4295
9	GO:0040017	positive regulation of locomotion	4.33E-06	2.27	63	361
10	GO:0051272	positive regulation of cellular component movement	1.78E-05	2.18	61	360

GO-terms associated to genes downregulated by ONECUT2

	ID	GO-term	p-value (FDR-adjusted)	Odds ratio	Associated differential genes	Total associated genes
1	GO:0036211	protein modification process	1.43E-11	1.46	750	3410
2	GO:0051674	localization of cell	1.14E-07	1.59	283	1154
3	GO:0048646	anatomical structure formation involved in morphogenesis	2.18E-06	1.58	232	946
4	GO:0072358	cardiovascular system development	2.21E-06	1.77	150	557
5	GO:0090132	epithelium migration	6.01E-06	2.31	70	214
6	GO:0033036	macromolecule localization	2.56E-05	1.32	537	2542
7	GO:0022008	neurogenesis	4.02E-05	1.56	185	763
8	GO:0043632	modification-dependent macromolecule catabolic process	1.06E-04	1.63	141	554
9	GO:0032956	regulation of actin cytoskeleton organization	1.28E-04	1.95	77	264
10	GO:0023051	regulation of signaling	1.28E-04	1.28	560	2707

GO-terms associated to genes downregulated by ONECUT3

	ID	GO-term	p-value (FDR-adjusted)	Odds ratio	Associated differential genes	Total associated genes
1	GO:0072358	cardiovascular system development	9.05E-13	2.48	124	539
2	GO:0048870	cell motility	8.66E-12	1.92	209	1129
3	GO:0001568	blood vessel development	8.74E-10	2.37	100	449
4	GO:0022603	regulation of anatomical structure morphogenesis	1.68E-09	1.92	161	857
5	GO:0090132	epithelium migration	3.82E-08	2.94	56	210
6	GO:0001655	urogenital system development	1.81E-07	2.50	67	284
7	GO:0048858	cell projection morphogenesis	8.64E-07	1.82	131	722
8	GO:0051271	negative regulation of cellular component movement	1.64E-06	2.59	54	222
9	GO:2000026	regulation of multicellular organismal development	3.65E-06	1.58	197	1251
10	GO:0040017	positive regulation of locomotion	3.71E-06	2.13	75	360

GO-terms associated to genes lower expressed in iNeurons than in fibroblasts

	ID	GO-term	p-value (FDR-adjusted)	Odds ratio	Associated differential genes	Total associated genes
1	GO:0072358	cardiovascular system development	9.19E-20	2.96	268	550
2	GO:0001568	blood vessel development	1.76E-15	2.97	218	446
3	GO:0051674	localization of cell	1.62E-12	1.99	446	1152
4	GO:0043207	response to external biotic stimulus	5.39E-08	2.31	248	578
5	GO:0071310	cellular response to organic substance	5.39E-08	1.87	421	1143
6	GO:0051272	positive regulation of cellular component movement	1.24E-05	2.62	171	370
7	GO:0044236	multicellular organism metabolic process	9.94E-04	6.46	58	85
8	GO:0009617	response to bacterium	9.77E-03	2.47	156	348
9	GO:0006950	response to stress	1.06E-02	1.91	275	764
10	GO:0090130	tissue migration	6.61E-01	2.88	104	213

Table S7. Annotation cell types ENCODE/ROADMAP samples

EID	Cell type	Lineage
E001	ES-I3 Cells	ESC
E002	ES-WA7 Cells	ESC
E003	H1 Cells	ESC
E004	H1 BMP4 Derived Mesendoderm Cultured Cells	ES-deriv
E005	H1 BMP4 Derived Trophoblast Cultured Cells	ES-deriv
E006	H1 Derived Mesenchymal Stem Cells	ES-deriv
E007	H1 Derived Neuronal Progenitor Cultured Cells	ES-deriv
E008	H9 Cells	ESC
E009	H9 Derived Neuronal Progenitor Cultured Cells	ES-deriv
E010	H9 Derived Neuron Cultured Cells	ES-deriv
E011	hESC Derived CD184+ Endoderm Cultured Cells	ES-deriv
E012	hESC Derived CD56+ Ectoderm Cultured Cells	ES-deriv
E013	hESC Derived CD56+ Mesoderm Cultured Cells	ES-deriv
E014	HUES48 Cells	ESC
E015	HUES6 Cells	ESC
E016	HUES64 Cells	ESC
E017	IMR90 fetal lung fibroblasts Cell Line	IMR90
E018	iPS-15b Cells	iPSC
E019	iPS-18 Cells	iPSC
E020	iPS-20b Cells	iPSC
E021	iPS DF 6.9 Cells	iPSC
E022	iPS DF 19.11 Cells	iPSC
E023	Mesenchymal Stem Cell Derived Adipocyte Cultured Cells	Mesench
E024	ES-UCSF4 Cells	ESC
E025	Adipose Derived Mesenchymal Stem Cell Cultured Cells	Mesench
E026	Bone Marrow Derived Cultured Mesenchymal Stem Cells	Mesench
E027	Breast Myoepithelial Primary Cells	Epithelial
E028	Breast variant Human Mammary Epithelial Cells (vHMEC)	Epithelial
E029	Primary monocytes from peripheral blood	HSC & B-cell
E030	Primary neutrophils from peripheral blood	HSC & B-cell
E031	Primary B cells from cord blood	HSC & B-cell
E032	Primary B cells from peripheral blood	HSC & B-cell
E033	Primary T cells from cord blood	Blood & T-cell
E034	Primary T cells from peripheral blood	Blood & T-cell
E035	Primary hematopoietic stem cells	HSC & B-cell
E036	Primary hematopoietic stem cells short term culture	HSC & B-cell
E037	Primary T helper memory cells from peripheral blood 2	Blood & T-cell
E038	Primary T helper naive cells from peripheral blood	Blood & T-cell
E039	Primary T helper naive cells from peripheral blood	Blood & T-cell
E040	Primary T helper memory cells from peripheral blood 1	Blood & T-cell
E041	Primary T helper cells PMA-I stimulated	Blood & T-cell
E042	Primary T helper 17 cells PMA-I stimulated	Blood & T-cell
E043	Primary T helper cells from peripheral blood	Blood & T-cell
E044	Primary T regulatory cells from peripheral blood	Blood & T-cell
E045	Primary T cells effector/memory enriched from peripheral blood	Blood & T-cell
E046	Primary Natural Killer cells from peripheral blood	HSC & B-cell
E047	Primary T CD8+ naive cells from peripheral blood	Blood & T-cell
E048	Primary T CD8+ memory cells from peripheral blood	Blood & T-cell
E049	Mesenchymal Stem Cell Derived Chondrocyte Cultured Cells	Mesench
E050	Primary hematopoietic stem cells G-CSF-mobilized Female	HSC & B-cell
E051	Primary hematopoietic stem cells G-CSF-mobilized Male	HSC & B-cell
E052	Muscle Satellite Cultured Cells	Myosat
E053	Cortex derived primary cultured neurospheres	Neurosph
E054	Ganglion Eminence derived primary cultured neurospheres	Neurosph
E055	Foreskin Fibroblast Primary Cells skin01	Epithelial
E056	Foreskin Fibroblast Primary Cells skin02	Epithelial
E057	Foreskin Keratinocyte Primary Cells skin02	Epithelial
E058	Foreskin Keratinocyte Primary Cells skin03	Epithelial
E059	Foreskin Melanocyte Primary Cells skin01	Epithelial
E061	Foreskin Melanocyte Primary Cells skin03	Epithelial
E062	Primary mononuclear cells from peripheral blood	Blood & T-cell
E063	Adipose Nuclei	Adipose
E065	Aorta	Heart
E066	Liver	Other
E067	Brain Angular Gyrus	Brain
E068	Brain Anterior Caudate	Brain
E069	Brain Cingulate Gyrus	Brain
E070	Brain Germinal Matrix	Brain
E071	Brain Hippocampus Middle	Brain
E072	Brain Inferior Temporal Lobe	Brain
E073	Brain_Dorsolateral_Prefrontal_Cortex	Brain
E074	Brain Substantia Nigra	Brain

ENCODE ID	Cell type	Lineage
E075	Colonic Mucosa	Digestive
E076	Colon Smooth Muscle	Sm. Muscle
E077	Duodenum Mucosa	Digestive
E078	Duodenum Smooth Muscle	Sm. Muscle
E079	Esophagus	Digestive
E080	Fetal Adrenal Gland	Other
E081	Fetal Brain Male	Brain
E082	Fetal Brain Female	Brain
E083	Fetal Heart	Heart
E084	Fetal Intestine Large	Digestive
E085	Fetal Intestine Small	Digestive
E086	Fetal Kidney	Other
E087	Pancreatic Islets	Other
E088	Fetal Lung	Other
E089	Fetal Muscle Trunk	Muscle
E090	Fetal Muscle Leg	Muscle
E091	Placenta	Other
E092	Fetal Stomach	Digestive
E093	Fetal Thymus	Thymus
E094	Gastric	Digestive
E095	Left Ventricle	Heart
E096	Lung	Other
E097	Ovary	Other
E098	Pancreas	Other
E099	Placenta Amnion	Other
E100	Psoas Muscle	Muscle
E101	Rectal Mucosa Donor 29	Digestive
E102	Rectal Mucosa Donor 31	Digestive
E103	Rectal Smooth Muscle	Sm. Muscle
E104	Right Atrium	Heart
E105	Right Ventricle	Heart
E106	Sigmoid Colon	Digestive
E107	Skeletal Muscle Male	Muscle
E108	Skeletal Muscle Female	Muscle
E109	Small Intestine	Digestive
E110	Stomach Mucosa	Digestive
E111	Stomach Smooth Muscle	Sm. Muscle
E112	Thymus	Thymus
E113	Spleen	Other
E114	A549 EtOH 0.02pct Lung Carcinoma Cell Line	ENCODE2012
E115	Dnd41 TCell Leukemia Cell Line	ENCODE2012
E116	GM12878 Lymphoblastoid Cells	ENCODE2012
E117	HeLa-S3 Cervical Carcinoma Cell Line	ENCODE2012
E118	HepG2 Hepatocellular Carcinoma Cell Line	ENCODE2012
E119	HMEC Mammary Epithelial Primary Cells	ENCODE2012
E120	HSMM Skeletal Muscle Myoblasts Cells	ENCODE2012
E121	HSMM cell derived Skeletal Muscle Myotubes Cells	ENCODE2012
E122	HUVEC Umbilical Vein Endothelial Primary Cells	ENCODE2012
E123	K562 Leukemia Cells	ENCODE2012
E124	Monocytes-CD14+ RO01746 Primary Cells	ENCODE2012
E125	NH-A Astrocytes Primary Cells	ENCODE2012
E126	NHDF-Ad Adult Dermal Fibroblast Primary Cells	ENCODE2012
E127	NHEK-Epidermal Keratinocyte Primary Cells	ENCODE2012
E128	NHLF Lung Fibroblast Primary Cells	ENCODE2012
E129	Osteoblast Primary Cells	ENCODE2012

Map2 staining iNeurons

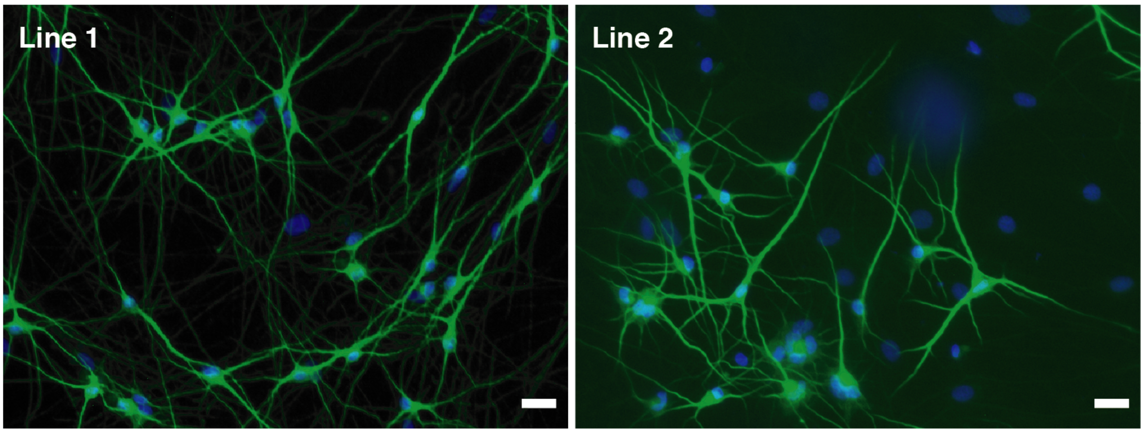

Figure S1. iNeuron Map2 staining.
MAP2 staining (green) in combination with nuclei staining (blue) of the iNeurons after 19 days of differentiation. Images are shown for the iNeurons derived from the 2 different iPSC cell lines used. Scale bars represent 20 µm.

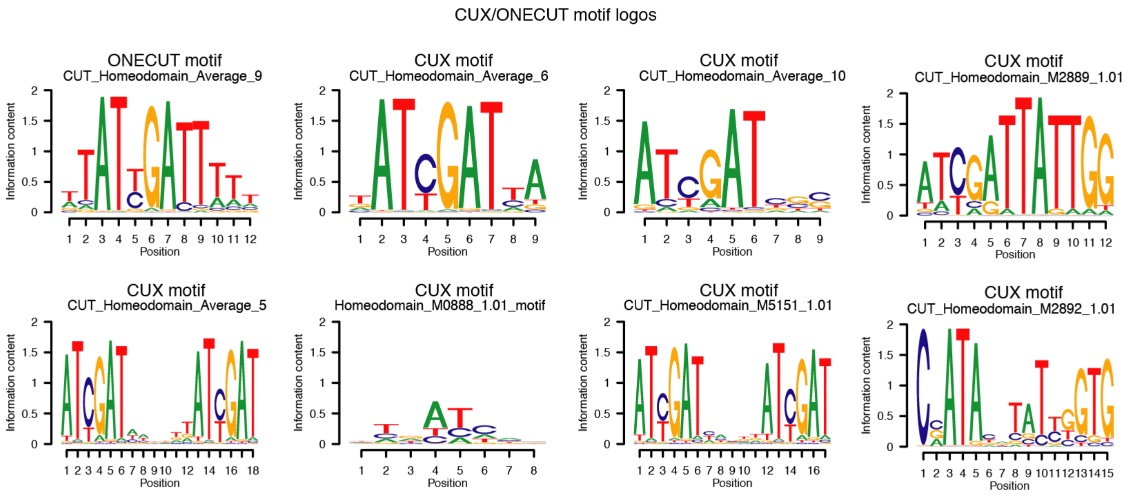

Figure S2. Position-weight-matrix logos.

Position weight matrix-based logos for the ONECUT and CUX transcription factors associated motifs (based on cis-bp database (1)).

a

Estimated MOI (mean±standard deviation)
for the lentiviral transductions

	Morphology / ATAC / RNA
Bclxl	5.3±1.6
ONECUT1	1.2±0.4
ONECUT2	1.7±0.6
ONECUT3	6.2±3.2

b

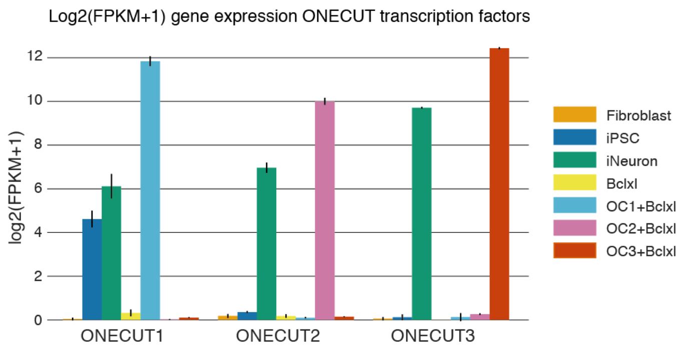

Figure S3. Transgene expression

a. Estimated mean and standard deviation of the multiplicity of infection (MOI) used for overexpression of Bclxl, OC1, OC2 and OC3 in morphology, ATAC-seq and RNA-seq validation experiments.

b. Log2(FPKM+1) gene expression (RNA-seq) for ONECUT1, ONECUT2 and ONECUT3 in fibroblasts, iPSCs, iNeurons, Bclxl, OC1+Bclxl, OC2+Bclxl and OC3+Bclxl. Shown are the mean and standard deviation calculated over all replicates (both biological and technical).

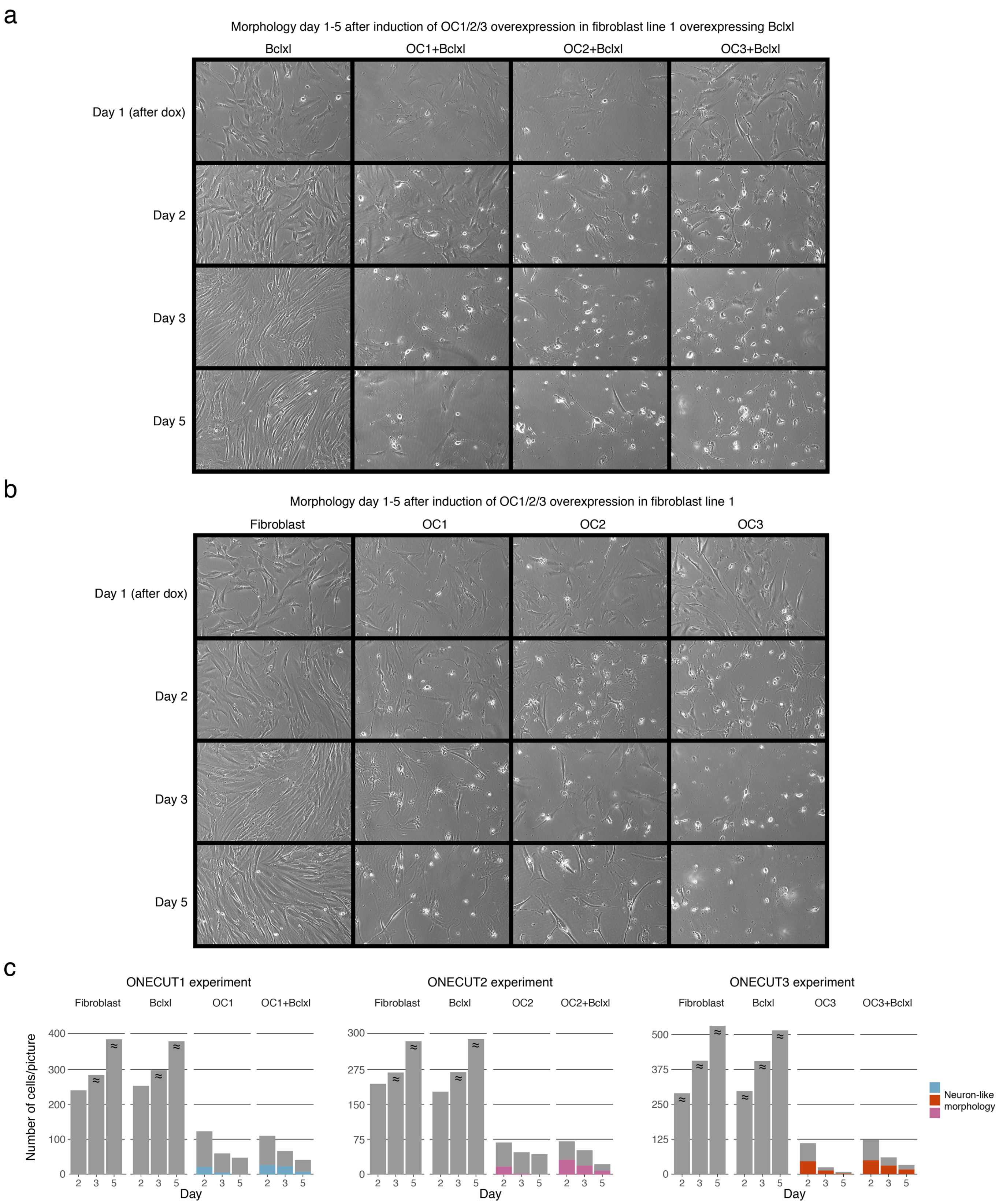

Figure S4. Cell morphology at day 1-5 after overexpression of OC1/2/3.

a. Morphology on day 1-5 after induction of OC1/2/3 overexpression in adult human fibroblast line 1.

b. Morphology on day 1-5 after induction of OC1/2/3 overexpression in adult human fibroblast line 1 overexpressing Bclxl.

c. Cell number quantification on day 2, 3 and 5 after doxycycline induction. The number of cells and the number of neuron-like cells were quantified for three separate experiments (ONECUT1, ONECUT2, ONECUT3) for which examples images are shown in panel **a** and **b**. For each condition (Fibroblast, Bclxl, OC1/2/3, OC1/2/3+Bclxl) four brightfield images were quantified and summed. The ≈ indicates cell numbers that were not counted but estimated based on cell density.

a

Quantification of the percentage of cells with neuron-like morphological characteristics on day 2

	Bclxl	OC1+Bclxl				OC2+Bclxl				OC3+Bclxl			
Cell line	%	%	$SD_{\%}$	$N_{Pictures}$	N_{cells}	%	$SD_{\%}$	$N_{Pictures}$	N_{cells}	%	$SD_{\%}$	$N_{Pictures}$	N_{cells}
1	0	26	5	9	853	45	6	6	352	44	7	6	564
2	0	42	10	5	409	50	7	5	343	70	9	6	268
3	0	26	7	6	537	47	6	6	437	67	7	6	300

b

Examples of images used for quantification of the percentage of cells with neuron-like morphological characteristics on day 2

1 = flat 'fibroblast-like' morphology

2 = neuron-like morphological characteristics

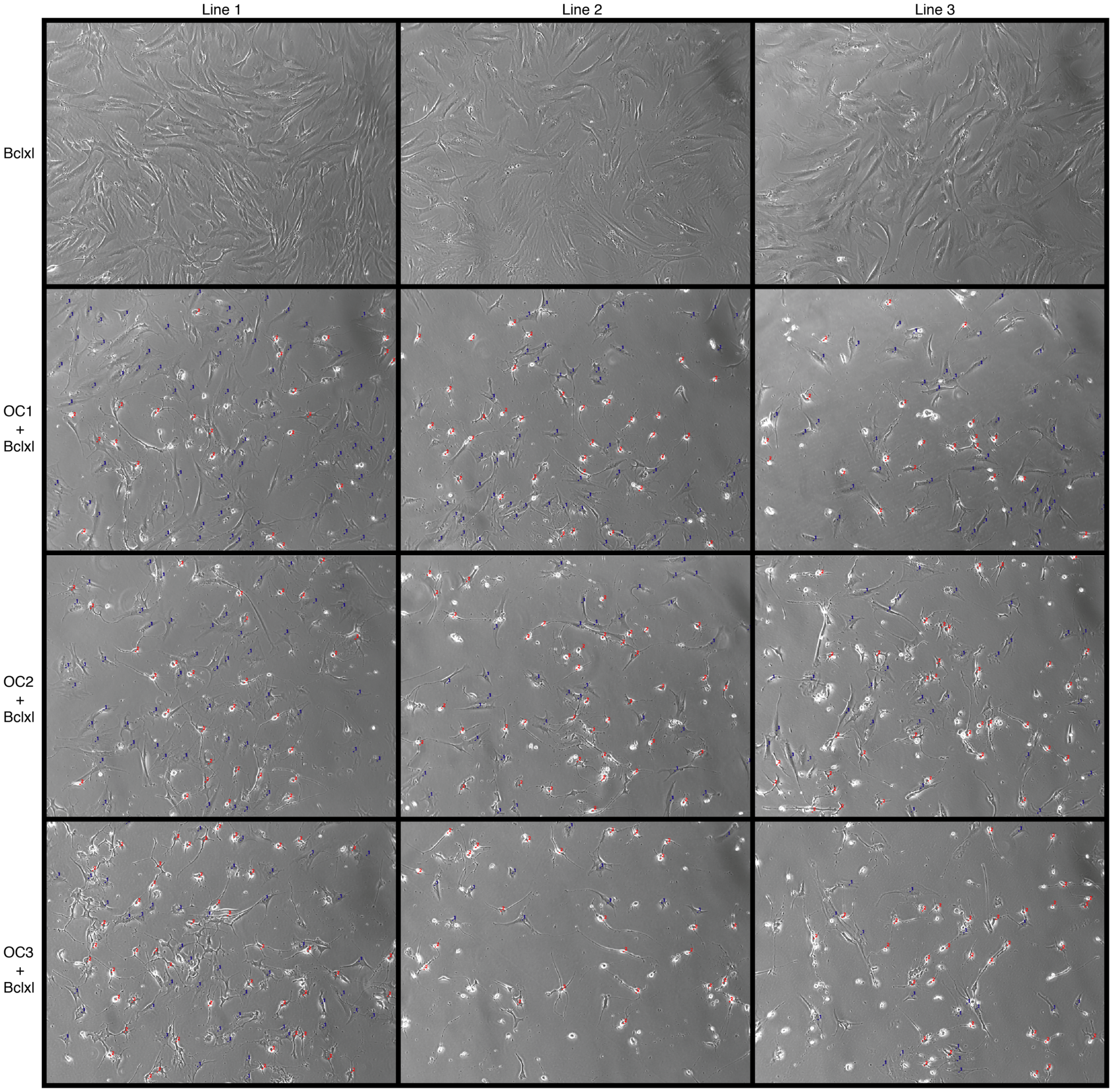

Figure S5. Quantification

a. Details of the quantification of the percentage of differentiating cells on day 2 after OC1/2/3 induction. $SD_{\%}$ is standard deviation of the percentage. $N_{Pictures}$ is the number of brightfield images used for the quantification. N_{cells} is the total number of cells quantified.

b. Example images (one for each condition and cell lines) of the quantification, showing which cells were assigned as flat fibroblast-like cells (1 in images) and which cells were assigned as differentiating cells (2 in images).

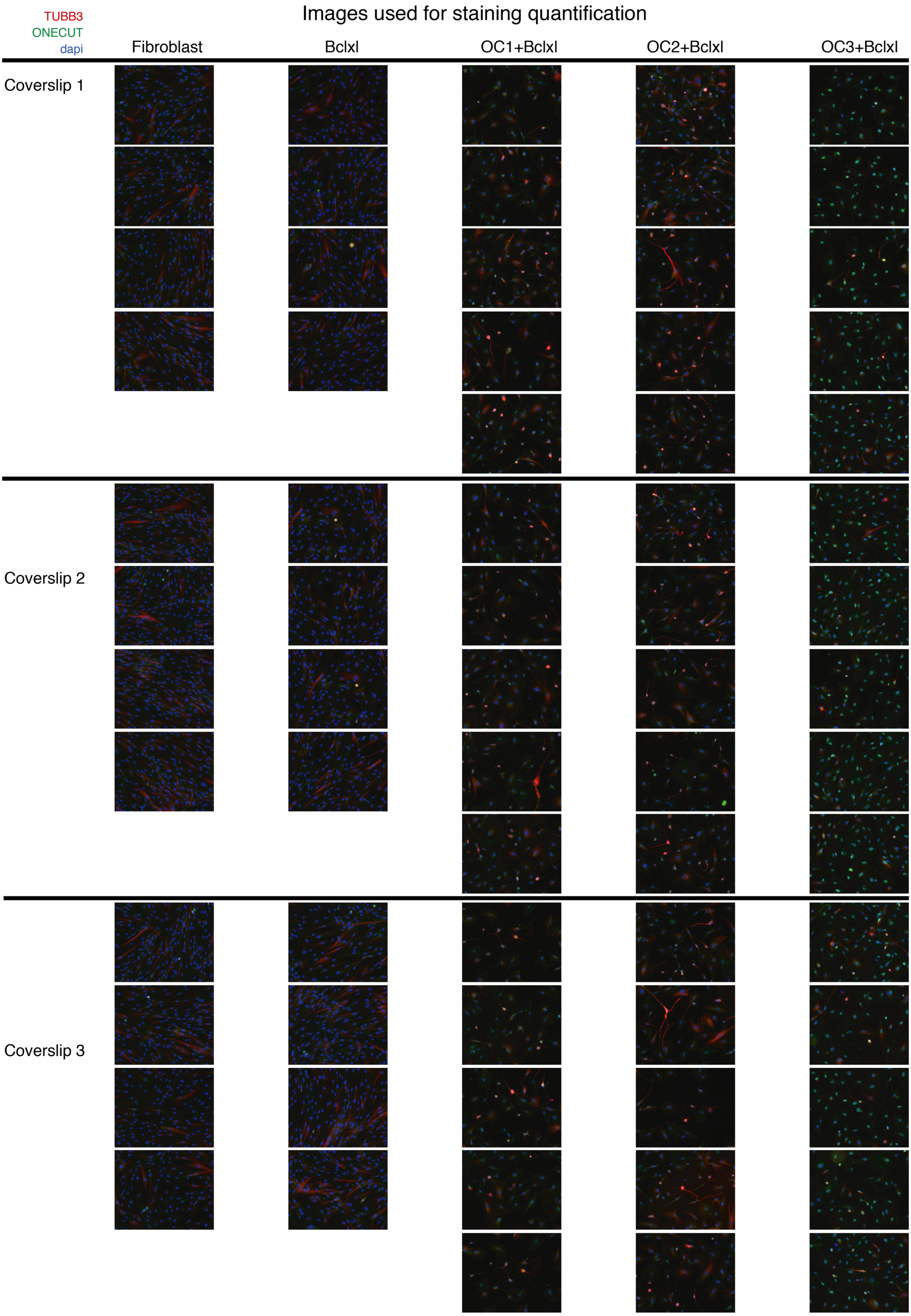

(Figure S6 legend on the next page)

Figure S6. Images used for staining quantification

All images used for the staining quantification. Per condition 3 coverslips were quantified, with 4-5 images per condition. On the images, TUBB3 is in red, ONECUT is in green and dapi is in blue. All images were taken with the same exposure times.

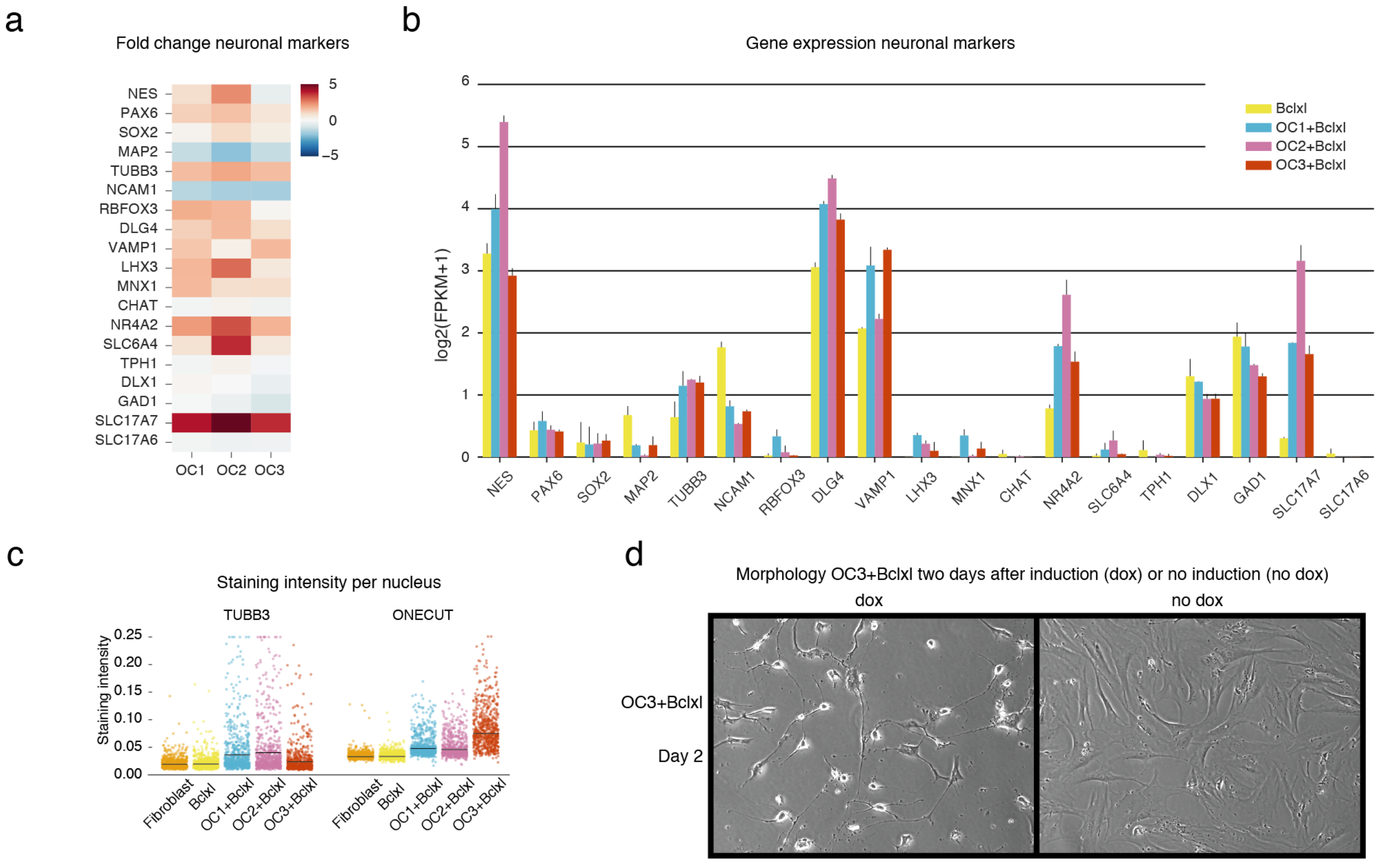

Figure S7. Neuronal marker expression

a. RNA-seq log2(FoldChange) following OC1/2/3 overexpression for the selected neuronal marker genes.

b. RNA-seq log2(FPKM+1) read counts in the Bclxl, OC1+Bclxl, OC2+Bclxl and OC3+Bclxl conditions, for the selected neuronal marker genes. Shown are mean and standard deviation for the two technical replicates.

c. Quantification of the mean staining intensity at individual nuclei for TUBB3 and ONECUT. Shown are 668 nuclei per condition. The average mean staining intensity over all 668 nuclei is represented by a horizontal line black line.

d. Morphology OC3+Bclxl on day 2 after induction (dox) or no induction (no dox) of OC3 overexpression.

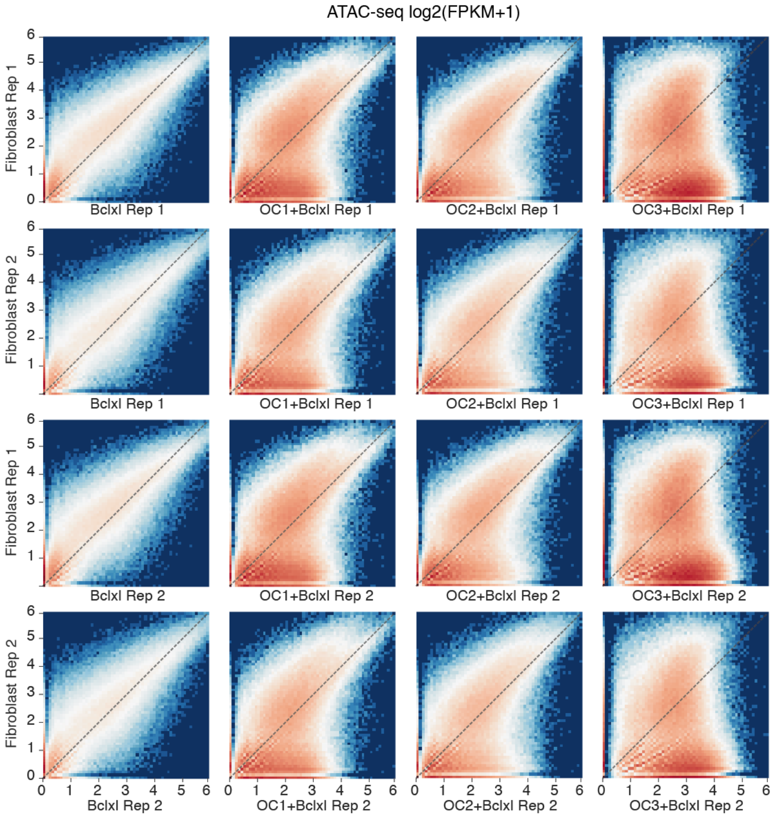

Figure S8. Chromatin accessibility changes induced by OC1/2/3 overexpression.

2D histogram of log2-transformed ATAC-seq fragment counts at ATAC union CRs for cell line 1. Bclxl condition technical replicates on the y-axis. Fibroblasts, OC1+Bclxl, OC2+Bclxl, and OC3+Bclxl technical replicates on the x-axis. Compared to Fig. **3d**, technical replicates are plotted separately.

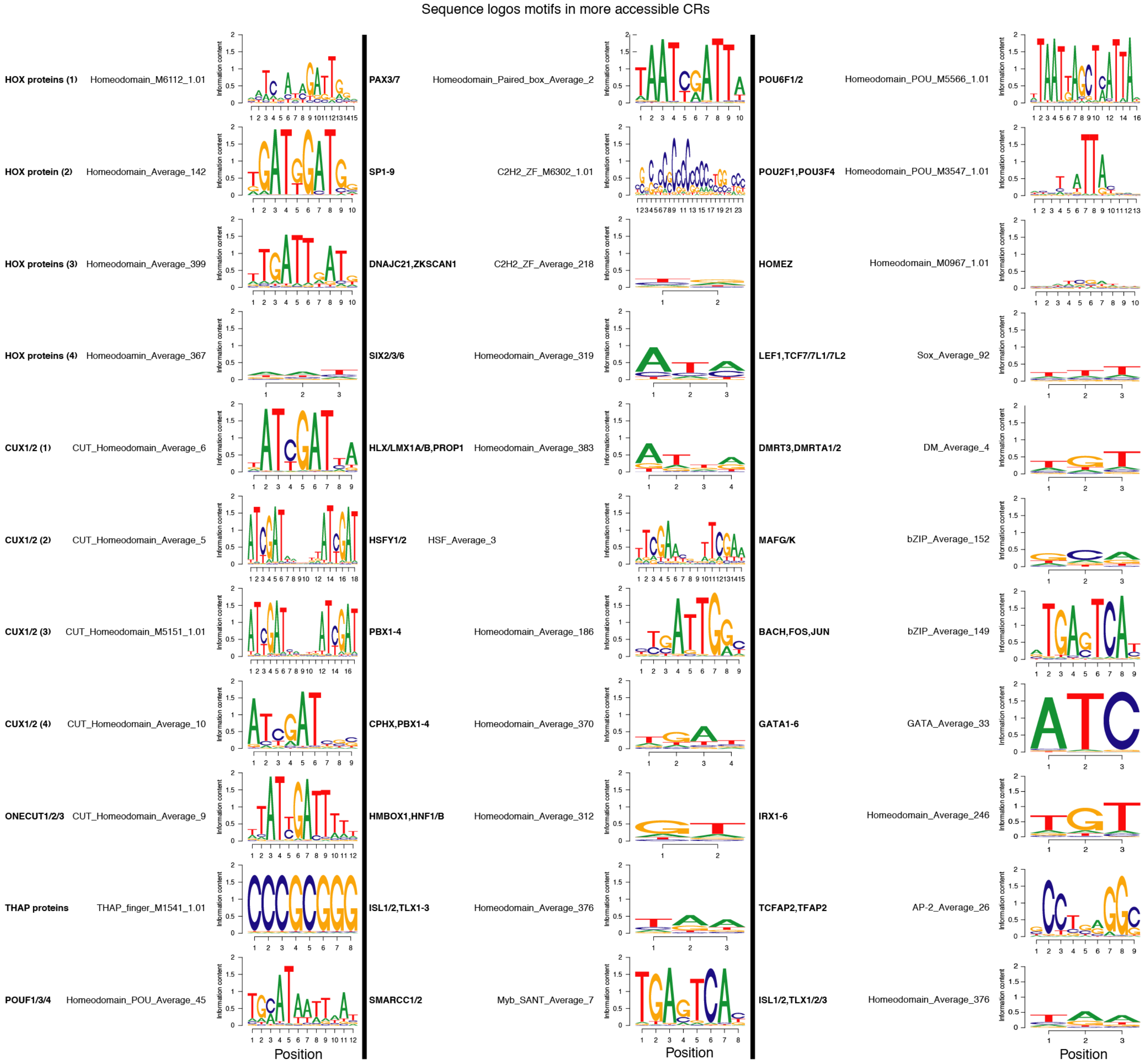

Figure S9. Motif logos for motifs enriched at OC1-up, OC2-up and OC3-up CRs.
Position weight matrix-based logos for the motifs associated with OC1-up, OC2-up and OC3-up CRs in Fig. 4j and Fig S10a.

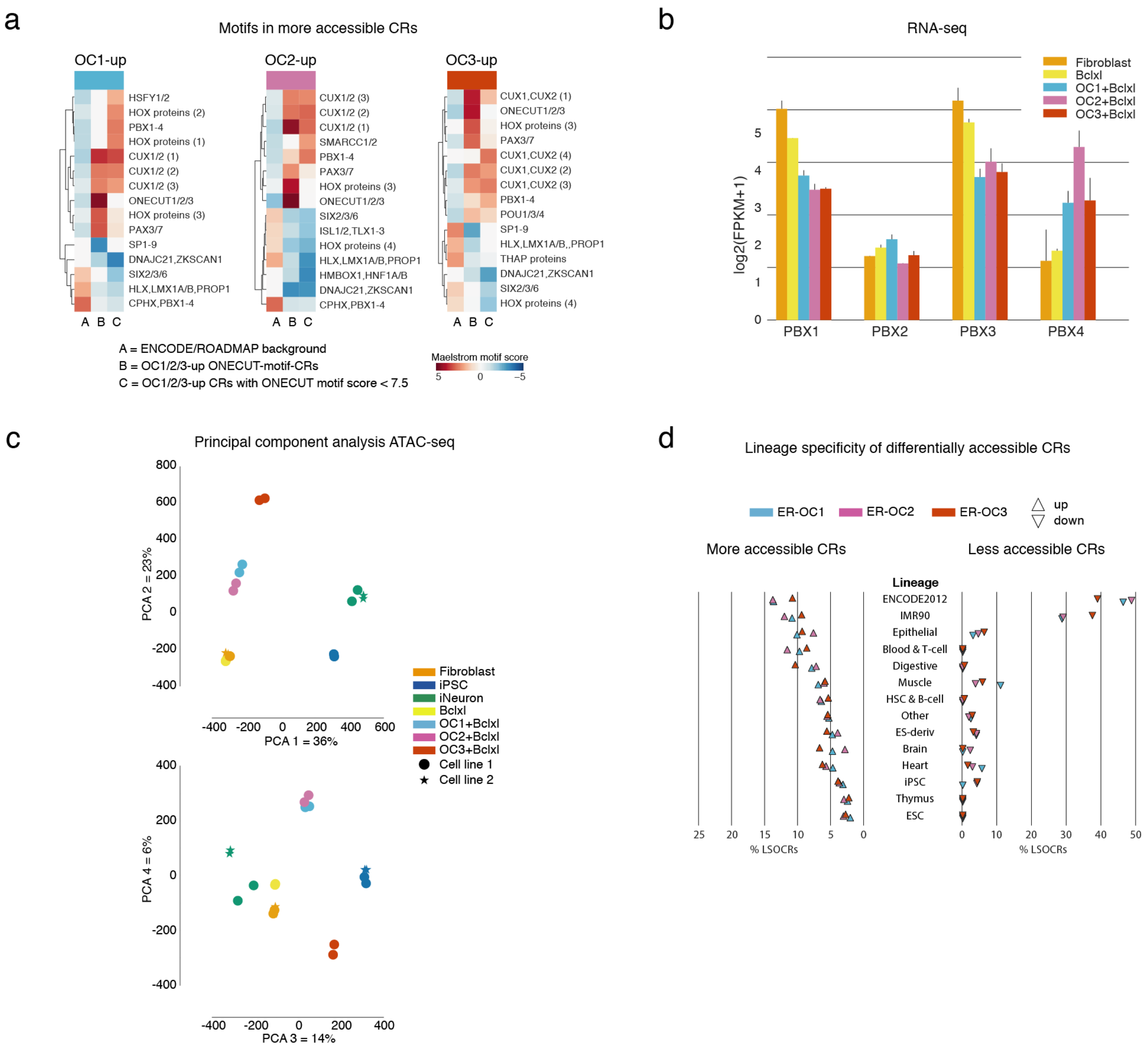

Figure S10. Enriched motifs and lineage specific chromatin regions

a. Enriched motifs in (ONECUT-motif-)CRs more accessible following OC/1/2/3 overexpresssion. Motifs determined using the GimmeMotifs function maelstrom, comparing an ENCODE/ROADMAP background CR set (A) to more accessible CRs with a ONECUT motif score >7.5 (B) and more accessible CRs with a ONECUT motif score < 2.5 (C). Motif logos in Fig. S9.

b. RNA-seq log2(FPKM+1) read counts in Fibroblast and the Bclxl, OC1+Bclxl, OC2+Bclxl and OC3+Bclxl conditions, for PBX1, PBX2, PBX3 and PBX4. Shown are mean and standard deviation for the two technical replicates for cell line 1.

c. Principal component analysis of ATAC-seq samples for the log2(FPKM+1) fragment count values determined on the ATAC union CR set.

d. Lineage specificity of differentially accessible CRs. Identical to Figure 5b, but without the iNeuron LSOCRs.

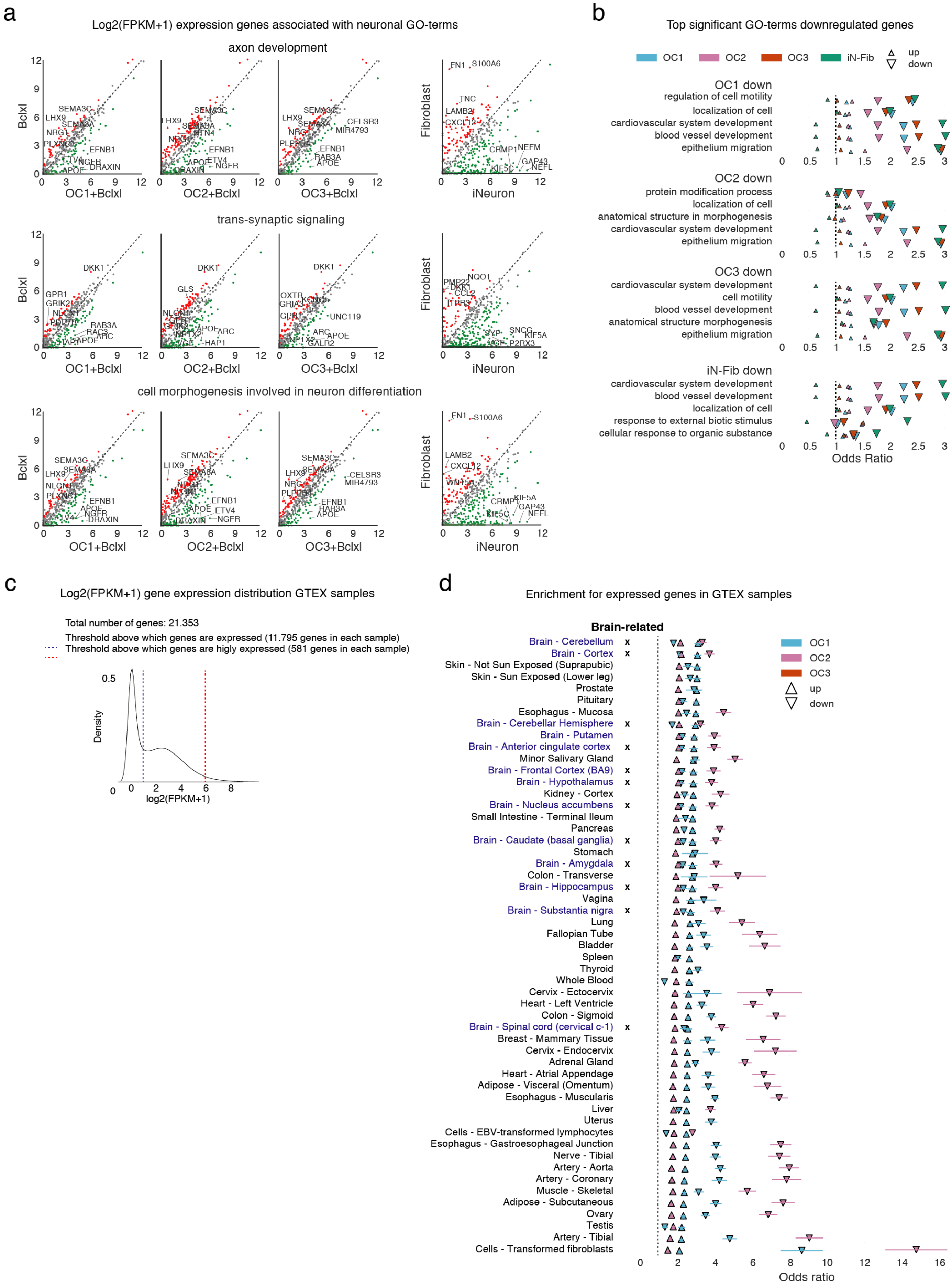

(Figure S11 legend on the next page)

Figure S11. GO-terms associated with downregulated genes and overlap with expressed GTEX genes.

a. Quantile normalized log2(FPKM+1) expression for genes associated with respectively the GO-terms 'axon development', 'trans-synaptic signaling' and 'cell morphogenesis involved in neuron differentiation'. The top five genes with the highest fold change are annotated for both upregulated (green) and downregulated genes (red).

b. GO-terms associated with differentially downregulated genes. Similar GO-terms were discarded based of information content in the GO-term graph (see Methods). For the five most significant GO-terms associated with respectively *OC1-down*, *OC2-down*, *OC3-down* and *iN-Fib-down*, the odds ratios are plotted for all differentially expressed gene sets (Fig. 6b).

c. Quantile normalized log2(FPKM+1) gene expression distribution for GTEX samples. The blue line indicates the threshold above which genes are expressed (panel d) and the red line the threshold above which genes are highly expressed (Fig. 6h).

d. Odds ratios for the overlap of differentially expressed gene sets (*OC1-up, OC2-up, OC3-up, OC1-down, OC2-down,OC3-down)* with genes expressed (log2(FPKM+1) > 1) in 8555 samples from the GTEX project (2). Plotted as triangles are the mean odd ratio and standard deviation for GTEX samples of the same tissue or cell type. The rows are ordered by the mean odds ratio for OC1. Brain-related GTEX samples are marked with an X.

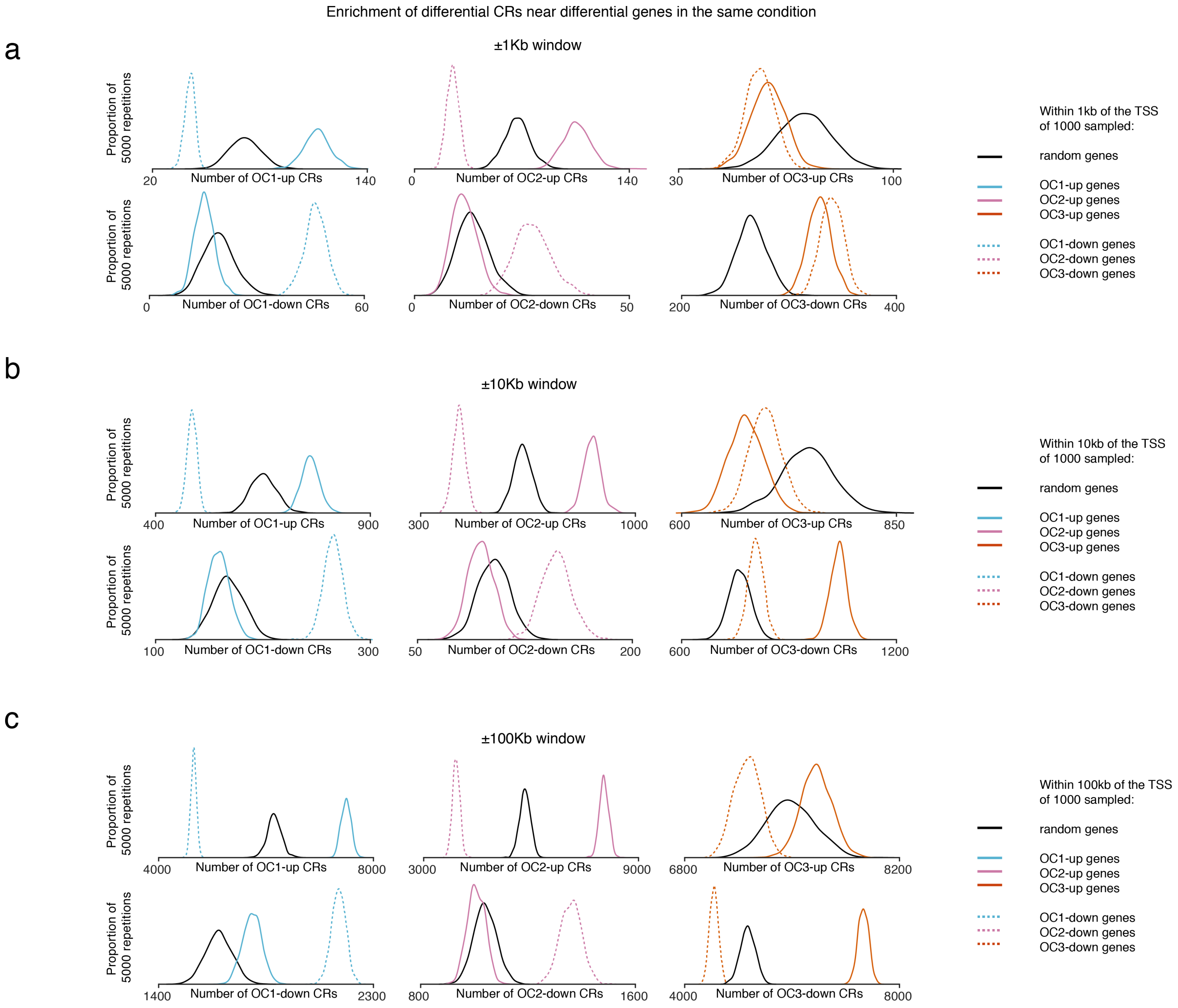

Figure S12. Enrichment of differential CRs near differential genes in the same condition

a. Enrichment within 1 kb of transcription start sites (TSSs). The figure shows distributions for the number of differential CRs (*OC1/2/3-up* or *OC1/2/3-down*) within 1 kb of the TSS of 1000 genes randomly sampled 5000 times. In each subpanel, distributions are plotted for three different gene sets from which the 1000 genes are sampled: 1) All GENCODE v.27 genes, 2) *OC-1/2/3-up* genes and 3) *OC1/2/3-down* genes.

b. Enrichment within 10 kb of transcription start sites (TSSs). Identical to panel **a**, but with a 10 kb distance.

c. Enrichment within 100 kb of transcription start sites (TSSs). Identical to panel **a**, but with a 100 kb distance.

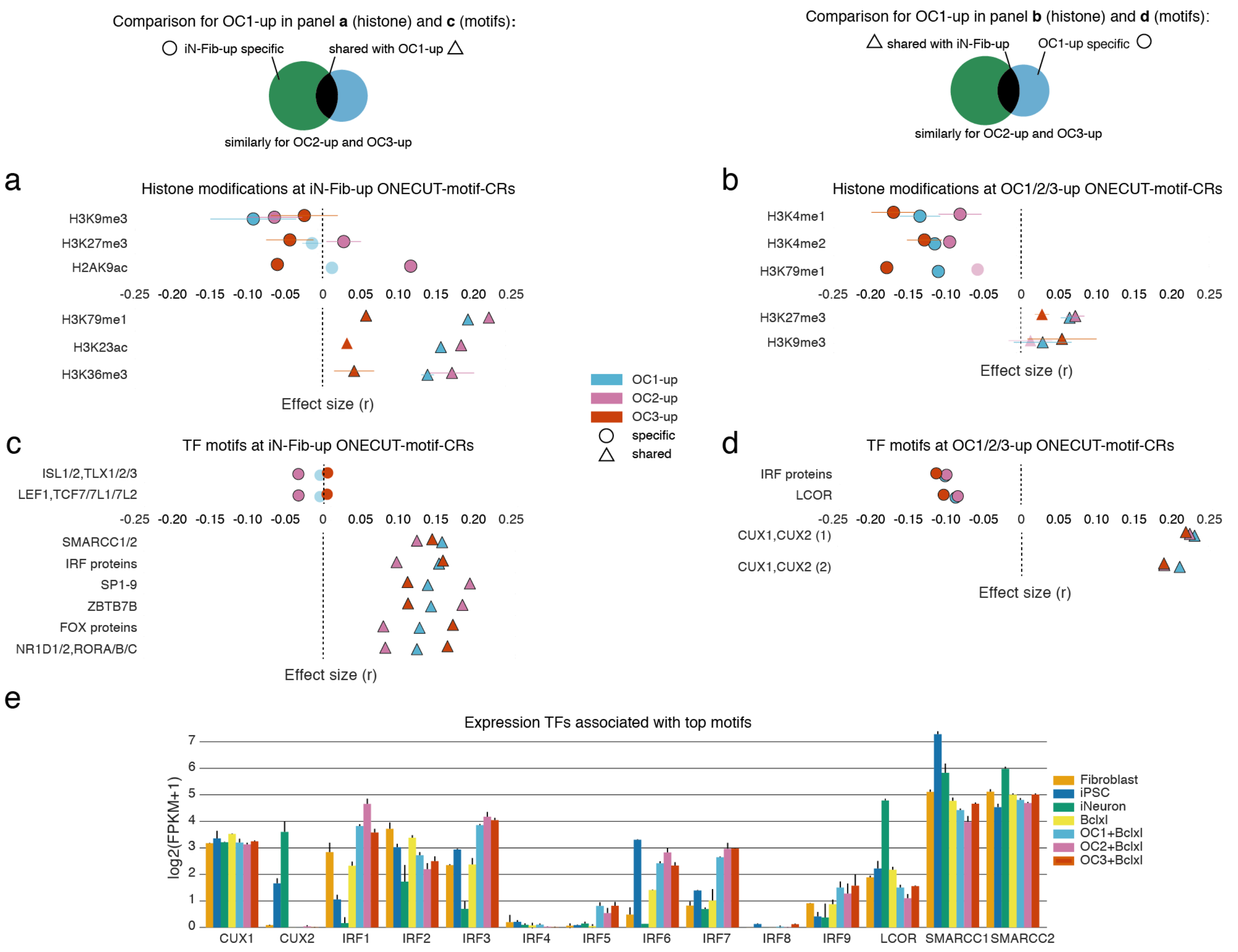

Figure S13. Histone modifications and TF motifs significantly associated with ONECUT-motif-CRs.

a. Histone modifications at ONECUT-motif-CRs specific for *iN-Fib-up* (circle) or shared with *OC1-up*, *OC2-up* or *OC3-up* (triangle). Benjamini Hochberg-adjusted p-values and effect sizes to quantify the shift in histone modification tag count across specific CRs relative to the shared CRs were calculated using a Mann-Whitney U test (see Methods). The top three significant (FDR-adjusted p-value < 0.01) histone modifications with the highest odds ratio for OC1-up, OC2-up and OC3-up were merged and plotted. The plotted confidence intervals indicate standard deviations over the ENCODE/ROADMAP fibroblast lines.

b. Histone modifications at ONECUT-motif-CRs specific *OC1-up, OC2-up or OC3-up,* or shared with *iN-Fib-up* (triangle). The analysis is identical to that in panel **a**, but for different CR sets.

c. Transcription factor motifs at ONECUT-motif-CRs specific for *iN-Fib-up* (circle) or shared with *OC1-up*, *OC2-up* or *OC3-up* (triangle). Benjamini Hochberg-adjusted p-values and effect sizes to quantify the shift in motif score distribution across specific CRs relative to the shared CRs were calculated using a Mann-Whitney U test (see Methods). The top three significant (FDR-adjusted p-value < 0.01) histone modifications with the highest odds ratio for OC1-up, OC2-up and OC3-up were merged and plotted. The plotted confidence intervals indicate standard deviations over the ENCODE/ROADMAP fibroblast lines.

d. Transcription factor motifs at ONECUT-motif-CRs specific *OC1-up, OC2-up or OC3-up,* or shared with *iN-Fib-up* (triangle). The analysis is identical to that in panel **c**, but for different CR sets.

e. Quantile normalized log2(FPKM+1) expression (RNA-seq) in fibroblasts, iPSCs, iNeurons, Bclxl, OC1+Bclxl, OC2+Bclxl and OC3+Bclxl, for transcription factors associated with the top motifs in panel **c** and **d**.

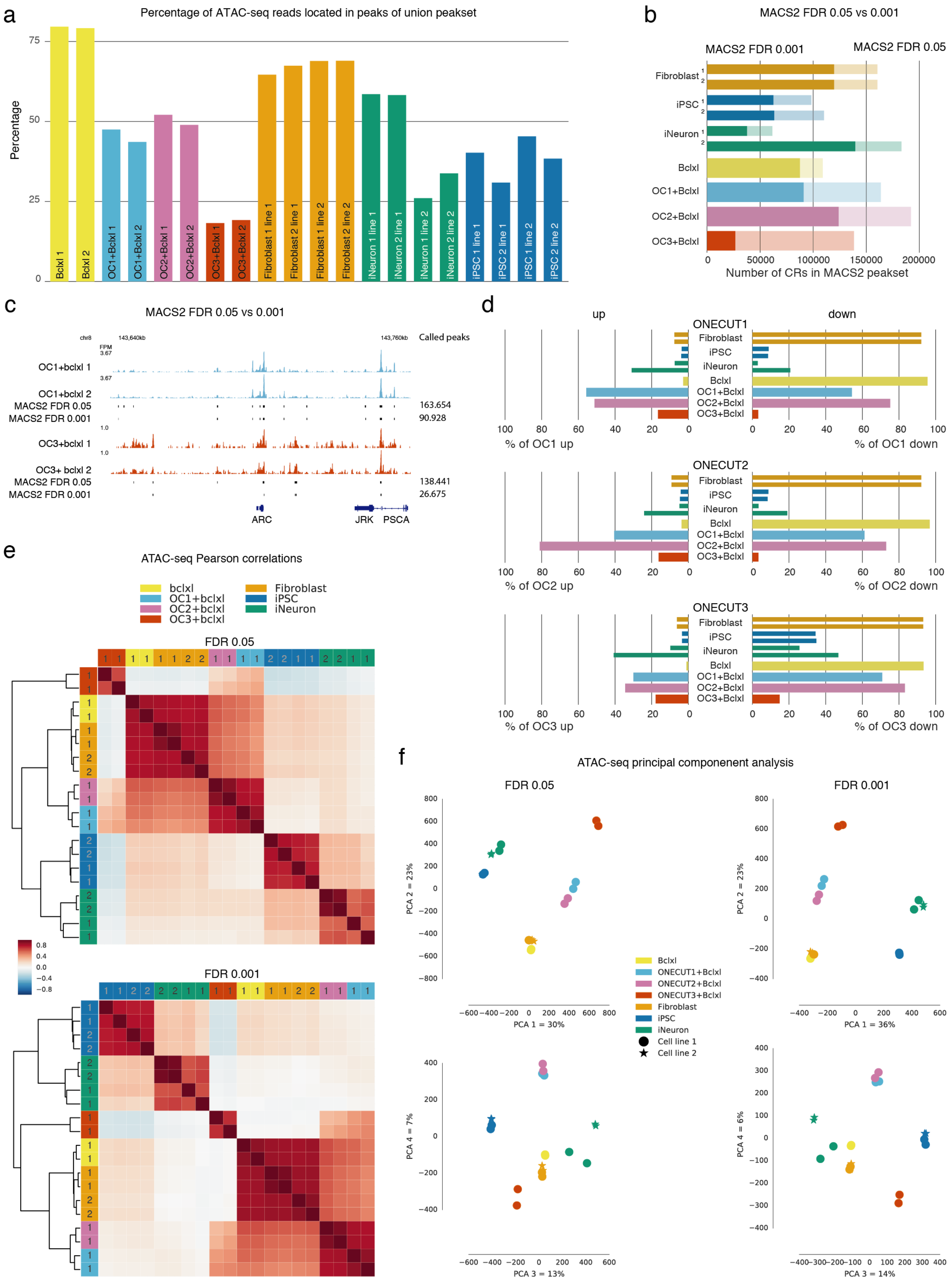

(Figure S14 legend on the next page)

Figure S14. ATAC-seq MACS2 peak calling using an FDR of 0.05 or 0.001

a. Percentage of filtered ATAC-seq reads that maps to a genomic region that is part of ATAC union CR set.

b. Number of CRs called by MACS2 using an FDR of 0.05 or 0.001.

c. Genomic region that illustrates the effect of FDR in MACS2 peak calling for OC3+Bclxl compared to OC1+Bclxl. FPM, fragments per million.

d. Percentage of CRs in differentially accessible CR sets (*OC1-up, OC2-up, OC3-up, OC1-down, OC2-down, OC3-down*) that overlaps with an OCR in the different OCR sets called by MACS2.

e. Hierarchical clustered heatmap of Pearson correlation coefficients of the quantile normalized log2(FPKM+1) ATAC-seq fragment counts, quantified on two different ATAC unions: created by merging peak sets determined using MACS with 1) an FDR on 0.05 and 2) an FDR of 0.001. Row and column colors indicate cell type, numbers inside the row and column colors indicate cell line.

f. Principle component analysis for the quantile normalized log2(FPKM+1) ATAC-seq fragment counts, quantified on two different ATAC unions: created by merging peak sets determined using MACS with 1) an FDR on 0.04 and 2) an FDR of 0.001.

Supplementary Materials and Methods

Cell culture

The human skin fibroblast lines were obtained from three anonymized healthy individuals and were stored at the Radboud University Medical Center Human Genetics biobank (Table S1). The use of the fibroblast lines was in accordance with the regulations of the Ethical Committee Arnhem-Nijmegen. The fibroblasts were cultured in tissue culture flasks (Corning) in Dulbecco's modified Eagle's medium containing 20 % (vol/vol) fetal bovine serum, 1 % (vol/vol) penicillin/streptomycin and 1 % (vol/vol) sodium pyruvate (all from Sigma-Aldrich), from here on referred to as fibroblast medium. Cells were regularly passaged using 0.25 % trypsin (BD Biosciences).

iPSC lines were obtained by lentiviral transduction of two of the fibroblast lines with the mouse OSKM (Oct4, Sox2, Klf4, Myc) cocktail. iPSC lines were cultured in 6 well plates coated with vitronectin (Gibco) in E8 medium (Gibco) containing 50 µg/mL G418 (Sigma-Aldrich) and 0.5 µg/mL puromycin (Sigma-Aldrich). iPSC cultures were regularly passaged using 0.5 mM UltraPure EDTA (Gibco).

Lentivirus production

In this study, multiple lentiviral transfer vectors were used (Table S2). In addition to the transfer vectors, we used two lentiviral packaging vectors for lentivirus production, psPAX2 (Addgene #12260) and pMD2.G (Addgene #12259). Lentivirus was produced by co-transfecting HEK293T cells with psPAX2, pMD2.G and the transfer vector using calcium phosphate co-precipitation. The HEK293T were incubated at 37 °C and 5% CO2 for 5-8 hours, after which the medium was replaced. 48 hours later, supernatant of the spent culture medium was collected. This supernatant was either first concentrated using an ultracentrifuge or directly stored at -80°C.

iNeuron differentiation

iNeuron differentiation was performed as described previously(3). Briefly, rtTA/Neurog2-positive iPSC lines were differentiated to iNeurons via doxycyclin-dependent Neurog2 overexpression over a period of three weeks (4). On day 21 after induction, cells were isolated for ATAC-seq and RNA-seq.

Validation experiments

The validation experiments consisted of overexpressing OC1/2/3 in human adult skin fibroblasts and were performed as follows. On day -2 (two days before induction of OC1/2/3 expression), cultured fibroblasts were detached using 0.25% trypsin, counted and resuspended in fibroblast medium. 20.000 fibroblasts were plated in 1ml fibroblast medium in each well of a twelve wells plate (Corning).

On day -1, cells were transduced with either only the Bclxl, OC1, OC2 or OC3 vector or the Bclxl vector in combination with the OC1, OC2 or OC3 vector. Transduction was performed in fresh fibroblast medium in the presence of 8ug/ml polybrene (Sigma-Aldrich). On day 0, 2 and 4, the medium was refreshed for medium containing 2ug/ml doxycycline (Sigma-Aldrich) to induce expression of the OC1/2/3 transgene. This medium also contained 2ug/ml puromycin (Sigma-Aldrich) for the Bclxl conditions and 8ug/ml blasticidin (Sigma-Aldrich) for the OC1/2/3 conditions. Morphology was assessed on day 0-5 using brightfield microscopy. One representative picture was taken for each well, with (at least) triplicates for each condition. For ATAC-seq and RNA-seq, cells were isolated on day 2.

Multiplicity of infection

For each batch of virus, the multiplicity of infection (MOI) was determined as follows. On day 0 we plated 20.000 cells of fibroblast line 1 into each well of a twelve well plate. On day 1 we transduced the cells with five different volumes (e.g. 0.25 µl, 0.5 µl, 1 µl, 2 µl and 4 µl) of virus in duplicates in the presence of 8 µg/ml polybrene (Sigma-Aldrich). Two wells were used as no virus control. On day 2, medium was refreshed for each well and 2 µg/ml doxycycline (Sigma-Aldrich) was added to induce transgene expression. On day 3 and 5, medium was refreshed for medium containing the 8 µg/ml of the selective agent blasticidin (Sigma-Aldrich). Finally, on day 7 the fraction of infected cells was assessed for each well by estimating the percentage of cell that had died due the selection, using a brightfield microscope.

To calculate a rough estimate of the MOI used in the validation experiments, we first averaged the estimated fraction of infected cells over the duplicates and applied a Poisson distribution (5) to estimate the MOI for 1 µl virus using the following equation: $MOI1ul = \frac{1}{n}\sum_{i=0}^{n} -\ln{(1-F_i)}\frac{1}{V_i}$

This equation averages over the five different virus volumes tested (n=5) and has as *F* the fraction of infected cells for condition *i* and as *V* the volume virus used in µl for condition *i*.

The MOI used in the validation experiment could then be determined by multiplying the MOI for 1 µl with the volume (µl) used in the validation experiment.

Efficiency quantification

Brightfield images were taken at random locations of the coverslip on day two after ONECUT induction. The set of images consisted of between five and nine images per condition per cell line. The morphology of each cell in these images was assessed by visual examination. We count the number of fibroblast-like flat cells and the number of stellate neuron-like or differentiating cells (example images with assigned cells in Fig. S5b). Cells that had neither a 'fibroblast-like' morphology nor neuron-like morphological changes were not included in the quantification. For each image we determined the percentage of stellate cells:

$$\%\ \text{stellate cells} = \frac{\text{number of stellate cells}}{(\text{number of flat cells} + \text{number of stellate cells})} * 100\%$$

The mean percentage and the standard deviation were calculated over the multiple images.

Immunohistochemistry

Cells were washed once with PBS and fixed in ice-cold 4% (mass/vol) paraformaldehyde in PBS at RT for 15 minutes. Fixed cells were washed three times with PBS, permeabilized with 0.2% Triton X-100 in PBS, washed three times with PBS and incubated with blocking buffer consisting of 10% (vol/vol) goat serum in PBS at RT for 1 hour. Next, cells were incubated with the primary antibodies rabbit anti-ONECUT3 (Biorbyt, orb312423, 1:100) and mouse anti-TUBB3 (Covance, MMS-435P, 1:1000) in 5% (vol/vol) goat serum in PBS at 4 °C overnight. After incubation, cells were washed three times with PBS, incubated with the conjugated secondary antibodies (Invitrogen) in 5% (vol/vol) goat serum in PBS at RT for 1 hour, washed three times with PBS, and incubated with 0.01% (vol/vol) Hoechst (Invitrogen) in PBS at RT for 10 minutes. Finally, cells were mounted onto slides with fluorescence mounting medium (Dako). Imaging was performed using an Axio Imager Z1 (Zeiss). CellProfiler (6) was used to quantify staining intensity for individual cells at the nucleus.

We should note that we stained for the overexpressed OC1/2/3 using a polyclonal antibody raised against a ONECUT3 epitope highly conserved in ONECUT1 (89% of a.a. sequence identical to epitope) and ONECUT2 (also 89% of a.a. sequence identical to epitope). The affinity of the antibody might not be the same for ONECUT1, ONECUT2 and ONECUT3. The staining intensities for OC1+Bclxl, OC2+Bclxl and OC3+Bclxl should therefore not be compared with each other, but only with the Bclxl condition.

ATAC-sequencing

iPSCs were harvested using accutase (Sigma-Aldrich) and fibroblasts using 0.25% trypsin (Gibco). Approximately 50,000 cells were collected and washed once with ice-cold PBS. Cells were centrifuged at 300 g and the supernatant was removed. iNeurons were not harvested, but 1-2 wells of the 12-well plate cultures (containing 50,000-100,000 cells) were washed once with ice-cold PBS. Isolation of the nuclei was performed by adding 500 µL lysis buffer (10 mM Tris/HCl [pH = 7.5], 10 mM NaCl, 3 mM $MgCl_2$ and 0.2 % [vol/vol] IGEPAL, in Milli-Q water/PBS [1:1]) to the cell pellet (iPSCs and fibroblasts) or to the cells on the plate (iNeurons), followed by mechanically dissociating the cells using a pipette. Nuclei were centrifuged at 500 g for 30 minutes. The pellet was washed once with ice-cold PBS and centrifuged at 500 g for 15 minutes. After removing most of the supernatant without disturbing the pellet, the nuclei were resuspended in a master mix of 10.5 µL nuclease-free water, 12.5 µL TD buffer and 2 µL Tn5 transposase (both Illumina Nextera DNA Library Prep kit) followed by incubation in a shaking heat block at 37 °C/650 rpm for 1 hour. After incubation, the reaction was cleaned by adding 5 µL clean-up buffer (0.9 M NaCl and 0.3 M EDTA [pH = 8] in nuclease-free water), 2 µL proteinase K (10 mg/mL) and 2 µL 5 % SDS. DNA was purified using normal-phase 2x AMPure bead purification (Beckman Coulter). The purified DNA was PCR amplified in 8 PCR cycles, followed by reverse-phase 0.55x AMPure bead purification (Beckman Coulter) and QIAquick column purification (Qiagen). The size-selected, purified PCR product was PCR amplified for another 8 PCR cycles, followed by QIAquick column purification (Qiagen). The fragment length distribution was determined using TapeStation (Agilent) and library concentration was quantified using KAPA library quantification kit (KAPA Biosystems). Sequencing was performed on an Illumina NextSeq 500 using HighOutput kit v2 for 75 cycles (paired-end 2x43 bp).

RNA-sequencing

RNA was isolated with the RNeasy Mini kit (Qiagen) according to the manufacturer's instructions. The RNA isolation included on-column DNA digestion. Before the RNA isolation, iPSCs and fibroblasts were lysed after harvesting the cells, whereas the iNeurons were lysed *in situ* (as described for ATAC-seq). RNA-seq library preparation was performed with the SMARTer Stranded Total RNA Sample Prep Kit (low input mammalian) (Clontech), according to the manufacturer's instructions. 50 ng of total RNA was depleted for rRNA using the RiboGone method included in the kit. The resulting rRNA-depleted RNA input was less than 10 ng and we followed the additional steps in the protocol of the manufacturer for this

amount of input material. 12 amplification cycles were used for the PCR. The fragment length distribution was determined using TapeStation (Agilent) and the last bead size selection of the protocol (with 1.0x beads) was repeated once more in case the libraries contained PCR product with sizes smaller than 100 bp. The library concentration was quantified using KAPA library quantification kit (KAPA Biosystems). Sequencing was performed on an Illumina NextSeq 500 using HighOutput kit v2 for 75 cycles (paired-end 2x43 bp).

Processing of sequencing data

ATAC-seq reads were aligned to a combined human-rat reference genome (respectively hg19 and rn6) using BWA sampe (7) with default parameters. All rn6-mapping reads and all h19-mapping reads with a mapping quality ≤ 40 were removed from the bam file (Number of mapped reads in Table S3). Peak calling was performed on the bam files using the MACS2 algorithm (8) (parameters: -f BAMPE -g hs --nomodel -q 0.001). Note that for the peak calling the two replicates of each cell line were taken together. Union CR sets were defined by combining all the peaks called for the different ATAC-seq samples and merging overlapping peaks to one peak. Quantification of the read counts on the union CR sets was performed using the R package GenomicAlignments (9) function summarizeOverlaps (parameters: mode = "Union", ignore.strand = TRUE, inter.feature = TRUE, singleEnd = FALSE, fragments = FALSE).

For RNA-seq, the first three bases of all forward reads were removed using the trimmomatic software (10) (parameters: SE HEADCROP:3), as recommended by the manufacturer of the SMARTer Stranded RNA Sample Prep Kit. Reads were mapped to the combined hg19-rn6 reference genome using STAR (11) with default parameters. All rn6-mapping reads and all h19-mapping reads with a mapping quality other than 255 were removed from the bam file (Number of mapped reads in Table S3). htseq-count (12) was used for quantifying the gene expression (parameters: --order=pos --stranded=yes --mode=union --type=exon --idattr=gene_id). For this, hg19 transcript definitions from GENCODE 27 (13) were used.

The resulting count tables for the ATAC-seq and RNA-seq (containing respectively the number of fragments per CR and gene) were used directly for differential analysis with DESeq2 (14) or were normalized to Fragments Per Kilobase Million (FPKM) values. The FPKM values were log2-transformed and quantile normalized in Python (https://github.com/ShawnLYU/Quantile_Normalize) to allow comparison between the different samples.

Union CR sets

We used three different union CR sets:

1) iNeuron-Fibroblast union: A reduced union CR set made by combining the iNeuron and fibroblast MACS2 peaksets and merging overlapping peaks to one peak.
2) ATAC union: A reduced union CR set made by combining the Bclxl, OC1+Bclxl, OC2+Bclxl, OC3+Bclxl, fibroblast, iPSC and iNeuron MACS2 peaksets and merging overlapping peaks to one peak.
3) DNase union: A union set of CRs with DNase I accessibility in at least one of 127 ENCODE/ROADMAP cell types. The DNase union was made by combining the CRs from the following three dataframes.
http://egg2.wustl.edu/roadmap/data/byDataType/dnase/state_calls_prom.RData
http://egg2.wustl.edu/roadmap/data/byDataType/dnase/state_calls_enh.RData
http://egg2.wustl.edu/roadmap/data/byDataType/dnase/state_calls_dyadic.RData

GimmeMotifs

The GimmeMotifs package (15) was used to predict transcription factor motifs on the different union CR sets. We used GimmeMotifs in three ways:

1) With a backgroundset, using 580 different transcription factor motifs coming from the 'gimme.vertebrate.v3.1.pwm' file supplied by GimmeMotifs or only the ONECUT motif in 'ONECUT_motif.pwm'. Applied to the different union CR sets, we used the following parameters and in this order:
gimme genome hg19; gimme index hg19; gimme background -n -g -i -l; gimme threshold gimme.vertebrate.v3.1.pwm/ONECUT_motif.pwm 0.01; gimme scan -g -p gimme.vertebrate.v3.1.pwm/ONECUT_motif.pwm -c. The GimmeMotifs output with a backgroundset and 580 motifs was used as input for the motif regression analysis. The GimmeMotifs output with a backgroundset and only the ONECUT motif was used to determine the ONECUT motif score threshold for ONECUT-motif-CRs.

2) Without a background set and using 580 different transcription factor motifs coming from the 'gimme.vertebrate.v3.1.pwm' file supplied by the GimmeMotifs suite or only the ONECUT motif in 'ONECUT_motif.pwm'. Applied to the different union CR sets, we used the following parameters: gimme scan -g hg19 gimme.vertebrate.v3.1.pwm/ONECUT_motif.pwm -T. This output was used to investigate the motif score distributions.

3) Comparing the DNase union CR set with the OC1-up, OC2-up and OC3-up CR sets to identify enriched motifs in the different CR sets. The OC1-up, OC2-up and OC3-up CR sets were

grouped into three different sets: CRs with a ONECUT motif score > 7.5, CRs with a ONECUT motif score < 7.5 and CRs with a ONECUT motif score < 2.5. We used the following parameters: gimme maelstrom input.txt hg19 output.
The motifs used in this study are supplied by GimmeMotifs and are motifs formed by clustering motifs in the cis-bp database (1) on similarity.

Differential chromatin accessibility and gene expression analysis

Both differential chromatin accessibility and differential gene expression were determined using DESeq2 (14). As input we used count values from the ATAC-seq/RNA-seq for CRs/genes. In all differential analyses with DESeq2, we used the design: *design ~ cell line + cell type*, to take into account both cell line and cell type. We considered CRs/genes differentially upregulated if the Benjamini-Hochberg-adjusted p-value < 0.01 and the log2(fold change) > 1. Similarly, we considered CRs/genes differentially downregulated if the Benjamini-Hochberg-adjusted p-value < 0.01 and the log2(fold change) < -1.

Using these criteria, we defined the following sets of differential accessible CRs: more accessible in OC1+Bclxl, OC2+Bclxl or OC3+Bclxl compared to Bclxl as respectively *OC1-up*, *OC2-up* and *OC3-up*; less accessible in OC1+Bclxl, OC2+Bclxl or OC3+Bclxl compared to Bclxl as respectively *OC1-down*, *OC2-down* and *OC3-down*; more accessible in iNeurons than in fibroblast as iN-Fib up; less accessible in iNeurons than in fibroblasts as iN-Fib down.

Using the same criteria, we defined the following sets of differentially expressed genes: higher expression in OC1+Bclxl, OC2+Bclxl or OC3+Bclxl compared to Bclxl as respectively *OC1-up*, *OC2-up* and *OC3-up*; lower expression in OC1+Bclxl, OC2+Bclxl or OC3+Bclxl compared to Bclxl as respectively *OC1-down*, *OC2-down* and *OC3-down*; higher expression in iNeurons than in fibroblast as iN-Fib-up; lower expression in iNeurons than in fibroblasts as iN-Fib-down.

GO-term enrichment analyses

GO-term enrichment analysis was performed for the differentially expressed gene sets (*OC1-up, OC2-up, OC3-up, iN-Fib-up, OC1-down, OC2-down, OC3-down, iN-Fib-down*), with one adjustment to these gene sets: to increase the size of the gene sets for the GO-term enrichment analysis, we used the less stringent Benjamini-Hochberg adjusted p-value < 0.1 as cutoff for significantly up- or downregulated genes from the DESeq2 analysis. We used the R module GOstats (16) to determine the enrichment of GO-terms in the different differentially expressed gene sets, using the GOstats hyperGTest (parameters: geneIds=EnsemblIDs in

DE_geneset, universeGeneIds=all EnsemblIDs in the DESeq2 results, annotation='org.Hs.eg.db', ontology='BP', pvalueCutoff=0.05, conditional=True, testDirection = 'over'). We used the GOstats summary function to obtain a table with the enriched GO-terms and the corresponding odds ratios and p-values. We restricted the analysis to Biological Process GO-terms and used a Benjamini-Hochberg-adjusted p-value < 0.01 as cutoff for significantly enriched GO-terms. Similar GO-terms were discarded based of information content in the GO-term graph. We used the GOstats function geneIdUniverse to obtain the genes (EnsemblIDs) annotated with each GO-term.

Regression analysis

We determined for each CR (denoted by *o*) in the iNeuron-Fibroblast union CR set, the change in FPKM values between iNeurons and fibroblasts (Δ_o = log2(1+FPKM(iNeuron CR *o*)) – log2(1+FPKM(fibroblast CR o))) and quantile normalized the distribution to a normal distribution with mean 0 and standard deviation 1. Both technical and biological replicates were averaged. We used GimmeMotifs (15) to generate for 580 clustered TF motifs based on the cis-bp database (1) binary motif calls, with $M \quad = 1$ for a predicted motif in a particular CR and $M \quad = 0$ for no predicted motif. To identify transcription factors associated with differential chromatin accessibility, we regressed differential chromatin accessibility Δ_o between iNeurons and fibroblasts on the presence of a transcription factor motif ${M_m}^o$, where *o* denotes the chromatin region *o* from the iNeuron-Fibroblast union set, and ${M_m}^o$= 1 denotes that motif *m* is present in CR *o.* We used a simple linear regression model $\Delta_o \quad = \mu + \beta_m {M_m}^o + \epsilon_o,\ \epsilon$ a with ϵ_o the residual, to estimate the regression coefficient β_m. To estimate the effect of a motif on differential accessibility while controlling for the effect if other motifs, we also used a multiple linear regression model $\Delta_o \quad = \mu + \beta_1 {M_1}^o + \beta_2 {M_2}^o + \cdots + \beta_{580} {M_{580}}^o + \epsilon_o$, where all motif regression coefficients are estimated simultaneously. We used a table linking motifs to 801 human transcription factors (produced using the cis-bp database 'http://cisbp.ccbr.utoronto.ca/' and the "gimme.vertebrate.v3.1.motif2factors.txt" file supplied by GimmeMotifs) to annotate motifs with the associated human transcription factors.

Venn diagrams

We intersected the different differentially accessible/expressed CR/gene sets (OC1-up, OC2 up, OC3-up, iN-Fib-up, OC1 down, OC2 down, OC3 down, iN-Fib down, as described above) and counted the number of CRs/genes in the different intersections. These numbers were

plotted as a Venn diagram using the http://eulerr.co/ web application (17). Areas are scaled (close to) proportional to the number of CRs.

Jaccard indices were determined using the following formula:

$$Jaccard\ index\ (A,B) = intersection(A,B)/union(A,B)$$

Overlap percentages were determined using the following formula:

$$Percentage(A) overlappedby(B) = intersection(A,B)/(A)\ *100\%$$

Fluff plots

We used the command line tool fluff (18) (parameters: heatmap -f -r -c -o) to produce for each differentially accessible CR set (*OC1-up, OC2-up, OC3-up, OC1-down, OC2-down, OC3-down*), a heatmap of the ATAC-seq coverage ±5 kilobase around around the centre of each CR. Each heatmap shows two technical replicates for both the Bclxl condition and either the OC1+Bclxl, OC2+Bclxl or OC3+Bclxl condition.

ONECUT-motif-OCRs comparison with ENCODE/ROADMAP

First, we adjusted the size of each CR in the ATAC union set and DNase union set (center CR±100bp) to prevent any bias due to CR size. We used GimmeMotifs (no background) to determine the ONECUT motif score for each CR in the ATAC union set and DNase union set and assigned the CRs with a motif score > 7.5 as ONECUT-motif-CRs (FPR = 0.01, determined using the command "gimme background -gc").

We obtained a dataframe with DNase I accessible CRs in the DNase union set for each of 127 ENCODE/ROADMAP cell types by combining the following dataframes:

http://egg2.wustl.edu/roadmap/data/byDataType/dnase/state_calls_prom.RData

http://egg2.wustl.edu/roadmap/data/byDataType/dnase/state_calls_enh.RData

http://egg2.wustl.edu/roadmap/data/byDataType/dnase/state_calls_dyadic.RData

This resulted in a dataframe with for each the DNase union set CR whether it is DNase I accessible for each of 127 ENCODE/ROADMAP cell types. We used bedtools (19) (parameters: intersect –a –b –c) to overlap each MACS2 peak set with the ATAC union CR set. This resulted in a table with for each CR in the ATAC union set whether it is present for of the MACS2 peak sets. For each ATAC-seq and ENCODE/ROADMAP sample we calculated the percentage of CRs that contained a ONECUT motif.

Overlap differentially accessible CRs with cell type OCRs and lineage specificity of differentially accessible CRs

First, we adjusted the size of each CR in the DNase union CR set (centre CR±100bp) to prevent any bias due to CR size. Next, we determined the coverage for both the ATAC-seq and 53 ENCODE/ROADMAP DNase-seq samples (downloaded from https://egg2.wustl.edu/roadmap/data/byFileType/alignments/consolidated/) on the DNase union.

The ATAC-seq coverage was quantified using the R package GenomicAlignments (9) function summarizeOverlaps (parameters: mode = "Union", ignore.strand = TRUE, inter.feature = TRUE, singleEnd = FALSE, fragments = FALSE). We used the "bedtools coverage" command to estimate the tag count for each DNase-seq sample in the ENCODE/ROADMAP consolidated data set. The ENCODE/ROADMAP consolidated data sets are downsampled to the same overall read depth and read length for each sample to avoid mapping bias.

Using the same criteria as described above for ATAC union CRs, we used DESeq2 to defined the following sets of differential accessible CRs in the DNase union CR set: more accessible in OC1+Bclxl, OC2+Bclxl or OC3+Bclxl compared to Bclxl as respectively *ER-OC1-up*, *ER-OC2-up* and *ER-OC3-up*; less accessible in OC1+Bclxl, OC2+Bclxl or OC3+Bclxl compared to Bclxl as respectively *ER-OC1-down*, *ER-OC2-down* and *ER-OC3-down*.

After quantile normalizing (https://github.com/ShawnLYU/Quantile_Normalize) the coverage to enable comparison between different samples, we used a threshold to determine for each CR whether it is accessible in a cell type or not. This threshold was the same for every sample, and chosen such that every sample had ~100,000 accessible CRs, which is similar to the number of peaks we call with MACS2 on the ATAC-seq data for one sample. Subsequently, we used bedtools (parameters: intersect –a –b –c) to overlap each differentially accessible CR set (*ER-OC1-up, ER-OC2-up, ER-OC3-up, ER-OC1-down, ER-OC2-down, ER-OC3-down*) with the DNase union CR set. This resulted in a table with for each CR in the DNase union set whether it is accessible for each DNase-seq and ATAC-seq sample, and whether it is present for each differentially accessible CR set.

To determine the odds ratio for the overlap of a differentially accessible CR set a DNase-seq and ATAC-seq sample, we used a Fisher's Exact Test (R module stats.fisher_tests) to determine the odds ratio for the overlap of a differentially accessible CR set a DNase-seq and ATAC-seq sample. We calculated the odds ratio for this overlap. Odds ratio = (M/n)/(N/x), with

M = Total number of CRs in the DNase union – N

N = Number of CRs accessible DNase-seq or ATAC-seq sample

n = Number of CRs differential following OC1/2/3 overexpression – x

x = Number of CRs both accessible DNase-seq or ATAC-seq sample and differential following OC1/2/3 overexpression.

To quantify lineage specificity, we grouped ENCODE/ROADMAP DNase-seq samples on cell lineage (using the ENCODE/ROADMAP annotation data) and included the iNeuron ATAC-seq as a separate lineage. Next, we determined for each DNase union CR whether or not the CR is only accessible in samples of a specific lineage and marked these as lineage specific OCRs (LSOCRs). We then determined for each lineage the percentage of LSOCRs differentially accessible after OC1/2/3 overexpression (*ER-OC1-up, ER-OC2-up, ER-OC3-up, ER-OC1-down, ER-OC2-down, ER-OC3-down*) relative to the other lineages.

$$\% \, LSOCRs = \frac{number\ of\ differentially\ accessible\ LSOCRs\ in\ lineage}{total\, number\ of\ differentially\ accessible\ LSOCRs} * 100\%$$

Comparison with GTEX

For this analysis we used RPKM gene expression for 8555 RNA-seq samples from the GTEX project (2) in the file 'GTEx_Analysis_v6_RNA-seq_RNA-SeQCv1.1.8 _gene_rpkm.gct.gz' (downloaded from https://www.gtexportal.org/home/datasets). We log2 transformed and quantile normalized (R function normalize.quantiles) the GTEX gene expression data. This resulted in the same log2(FPKM+1) gene expression distribution (Fig. S11c) for each GTEX samples. We used a threshold of log2(FPKM+1) > 1 to identify expressed genes for each sample and log2(FPKM+1) > 6 to determine only the highly expressed genes. By overlapping these genes with the genes in the differentially expressed gene sets (*OC1-up, OC2-up, OC3-up, OC1-down, OC2-down, OC3-down*), we determined for each GTEX sample the number of (highly) expressed genes that are also differentially expressed after OC1/2/3 overexpression. We calculated the odds ratio for this overlap using the following equation: Odds ratio = (M/n)/(N/x), with

M = Total number of genes (21.353) – N

N = Number of genes (highly) expressed in GTEX sample

n = Number of genes differentially expressed after OC1/2/3 overexpression – x

x = Number of genes both (highly) expressed in GTEX sample and differentially expressed after OC1/2/3 overexpression.

Using the GTEX annotation file 'GTEx_Data_V6_Annotations_SampleAttributesDS.txt' (downloaded from https://www.gtexportal.org/home/datasets), we calculated a mean odds ratio and standard deviation for each tissue type within the 8555 GTEX samples.

Enrichment induced CRs near induced genes

For each of OC1, OC2 and OC3, we performed the following analysis.

First, we sampled 1000 random genes 5000 times for each of the following gene sets:

- GENCODE 27 genes (random gene set)
- OC1/2/3-up genes
- OC1/2/3-down genes

Second, for each sample of 1000 genes, we counted in a ±1kb, ±10kb and ±100kb window around the transcription start site (TSS), the occurrence of the following CRs:

- OC1/2/3-up CRs
- OC1/2/3-down CRs

To overlap CRs and the different TSS windows, we used bedtools intersect -wao.

Three, we plotted the different counts for the 5000 permutations as a densityplot, always comparing the different gene sets in one plot.

Histone modifications and motifs associated with sets of differentially accessible CRs

We determined the coverage on the DNase union set for 25 different histone modifications, each one assayed in up to five ENCODE/ROADMAP fibroblast samples (E017, E055, E056, E126 and E128; Table S7). In addition, we called motif scores for 580 clustered TF motifs based on the cis-bp database (1) using GimmeMotifs (15). Both the histone modification coverage and motif scores were quantile normalized to a normal distribution with mean 0 and standard deviation 1.

We used bedtools (parameters: intersect –a –b –c) to overlap each differentially more accessible CR set (*OC1-up, OC2-up, OC3-up, iN-Fib-up*) with the DNase union CR set. We intersected the *iN-Fib-up* and *OC1/2/3-up* CR sets to get both the CRs shared and specific for the different CR sets. We used a threshold requiring the ONECUT motif score > 7.5 to select the ONECUT-motif-CRs (FPR=0.01, based on the background set) within the DNase union set.

For both histone modifications and motifs, we use a Mann-Whitney U-test (Python module scipy.stats.mannwhitneyu) to compare the distributions for *iN-Fib-up* specific to those shared with *OC1/2/3-up*, and *OC1/2/3-up* specific to those shared with *iN-Fib-up*. For the histone modifications, we calculated the mean and standard deviations for the effect size for each histone modification over the different fibroblast lines. We used Fisher's method to combine p-values over the different fibroblast lines (Pyhon module scipy.stats.combine_pvalues).

Pearson correlation heatmaps

Pearson correlations between the quantile normalized log2(FPKM+1) RNA-seq/ATAC-seq samples were calculated using the Python function .corr(method='pearson'). These correlations were plotted as a heatmap hierarchically clustered on Euclidean distance (clustermap) using the Python module Seaborn (https://seaborn.pydata.org/).

Web resources:

- GTEx data: http://www.gtexportal.org/static/datasets/gtex_analysis_v6
- Processed Roadmap DNase-seq data: https://personal.broadinstitute.org/meuleman/reg2map/HoneyBadger2_release/DNase/p2
- ENCODE/ROADMAP consolidated data set: http://egg2.wustl.edu/roadmap/data/byFileType/alignments/consolidated
- Quantile normalization: https://github.com/ShawnLYU/Quantile_Normalize
- Venn diagrams: http://eulerr.co
- GimmeMotifs: https://github.com/simonvh/gimmemotifs
- Bedtools: http://bedtools.readthedocs.io/en/latest

References

1. Weirauch,M.T., Yang,A., Albu,M., Cote,A.G., Montenegro-Montero,A., Drewe,P., Najafabadi,H.S., Lambert,S.A., Mann,I., Cook,K., *et al.* (2014) Determination and Inference of Eukaryotic Transcription Factor Sequence Specificity. *Cell*, **158**, 1431–1443.

2. Lonsdale,J., Thomas,J., Salvatore,M., Phillips,R., Lo,E., Shad,S., Hasz,R., Walters,G., Garcia,F., Young,N., *et al.* (2013) The Genotype-Tissue Expression (GTEx) project. *Nature Genetics*, **45**, 580–585.

3. Frega,M., van Gestel,S.H.C., Linda,K., van der Raadt,J., Keller,J., Van Rhijn,J.-R., Schubert,D., Albers,C.A. and Nadif Kasri,N. (2017) Rapid Neuronal Differentiation of Induced Pluripotent Stem Cells for Measuring Network Activity on Micro-electrode Arrays. *JoVE*, 10.3791/54900.

4. Zhang,Y., Pak,C., Han,Y., Ahlenius,H., Zhang,Z., Chanda,S., Marro,S., Patzke,C., Acuna,C., Covy,J., *et al.* (2013) Rapid Single-Step Induction of Functional Neurons from Human Pluripotent Stem Cells. *Neuron*, **78**, 785–798.

5. Ellis,E.L. and Delbrück,M. (1939) THE GROWTH OF BACTERIOPHAGE. *The Journal of General Physiology*, **22**, 365–384.

6. Carpenter,A.E., Jones,T.R., Lamprecht,M.R., Moffat,J., Gollan,P. and Sabatini,D.M. (2006) CellProfiler: image analysis software for identifying and quantifying cell phenotypes. *Genome Biology*, **7**, 100–110.

7. Li,H. and Durbin,R. (2010) Fast and accurate long-read alignment with Burrows–Wheeler transform. *Bioinformatics*, **26**, 589–595.

8. Zhang,Y., Liu,T., Meyer,C.A., Eeckhoute,J., Johnson,D.S., Bernstein,B.E., Nusbaum,C., Nyers,R.M., Brown,M., Li,W., *et al.* (2015) Model-based Analysis of ChIP-Seq (MACS). *Genome Biology*, **9**, 137–145.

9. Lawrence,M., Huber,W., Pagès,H., Aboyoun,P., Carlson,M., Gentleman,R., Morgan,M.T. and Carey,V.J. (2013) Software for Computing and Annotating Genomic Ranges. *PLoS Comput Biol*, **9**, e1003118–10.

10. Bolger,A.M., Lohse,M. and Usadel,B. (2014) Trimmomatic: a flexible trimmer for Illumina sequence data. *Bioinformatics*, **30**, 2114–2120.

11. Dobin,A. and Gingeras,T.R. (2002) Mapping RNA-seq Reads with STAR. . *Bioinformatics*, **29**, 15-21.

12. Anders,S., McCarthy,D.J., Chen,Y., Okoniewski,M., Smyth,G.K., Huber,W. and Robinson,M.D. (2013) Count-based differential expression analysis of RNA sequencing data using R and Bioconductor. *Nat Protoc*, **8**, 1765–1786.

13. Harrow,J., Frankish,A., Gonzalez,J.M., Tapanari,E., Diekhans,M., Kokocinski,F., Aken,B.L., Barrell,D., Zadissa,A., Searle,S., *et al.* (2012) GENCODE: The reference human genome annotation for The ENCODE Project. *Genome Research*, **22**, 1760–1774.

14. Love,M.I., Huber,W. and Anders,S. (2014) Moderated estimation of fold change and dispersion for RNA-seq data with DESeq2. *Genome Biology*, **15**, 31–21.

15. van Heeringen,S.J. and Veenstra,G.J.C. (2011) GimmeMotifs: a de novo motif prediction pipeline for ChIP-sequencing experiments. *Bioinformatics*, **27**, 270–271.

16. Falcon,S. and Gentleman,R. (2007) Using GOstats to test gene lists for GO term association. *Bioinformatics*, **23**, 257–258.

17. Larsson,J. (2018) Area-Proportional Euler and Venn Diagrams with Circles or Ellipses [R package eulerr version 4.1.0].

18. Georgiou,G. and van Heeringen,S.J. (2016) fluff: exploratory analysis and visualization of high-throughput sequencing data. *PeerJ*, **4**, e2209–10.

19. Quinlan,A.R. and Hall,I.M. (2010) BEDTools: a flexible suite of utilities for comparing genomic features. *Bioinformatics*, **26**, 841–842.
